# Enhanced Proteomic Coverage in Tissue Microenvironment by Immune Cell Subtype Library-Assisted DIA-MS

**DOI:** 10.1016/j.mcpro.2024.100792

**Published:** 2024-05-27

**Authors:** Jhih-Ci Yang, Tzi-Hui Hsu, Ciao-Syuan Chen, Jou-Hui Yu, Kuo-I Lin, Yu-Ju Chen

**Affiliations:** 1 Institute of Chemistry, Academia Sinica, Taipei, Taiwan; 2 Sustainable Chemical Science and Technology, Taiwan International Graduate Program, Academia Sinica and National Yang Ming Chiao Tung University, Taipei, Taiwan; 3 Department of Applied Chemistry, National Yang Ming Chiao Tung University, Hsinchu, Taiwan; 4 Genomics Research Center, Academia Sinica, Taipei, Taiwan; 5 Department of Chemistry, National Taiwan University, Taipei, Taiwan

**Keywords:** data-independent acquisition, proteomics, tumor microenvironment, colorectal cancer, mesenteric lymph node, tumor-infiltrating lymphocytes

## Abstract

Immune cells that infiltrate the tumor microenvironment (TME) play crucial roles in shaping cancer development and influencing clinical outcomes and therapeutic responses. However, obtaining a comprehensive proteomic snapshot of tumor-infiltrating immunity in clinical specimens is often hindered by small sample amounts and a low proportion of immune infiltrating cells in the TME. To enable in-depth and highly sensitive profiling of microscale tissues, we established an immune cell-enriched library-assisted strategy for data-independent acquisition mass spectrometry (DIA-MS). Firstly, six immune cell subtype-specific spectral libraries were established from sorted cluster of differentiation markers, CD8^+^, CD4^+^ T lymphocytes, B lymphocytes, natural killer cells, dendritic cells, and macrophages in murine mesenteric lymph nodes (MLNs), covering 7815 protein groups with surface markers and immune cell-enriched proteins. The feasibility of microscale immune proteomic profiling was demonstrated on 1 μg tissue protein from the tumor of murine colorectal cancer (CRC) models using single-shot DIA; the immune cell-enriched library increased coverage to quantify 7419 proteins compared to directDIA analysis (6978 proteins). The enhancement enabled the mapping of 841 immune function-related proteins and exclusive identification of many low-abundance immune proteins, such as CD1D1, and CD244, demonstrating high sensitivity for immune landscape profiling. This approach was used to characterize the MLNs in CRC models, aiming to elucidate the mechanism underlying their involvement in cancer development within the TME. Even with a low percentage of immune cell infiltration (0.25–3%) in the tumor, our results illuminate downregulation in the adaptive immune signaling pathways (such as C-type lectin receptor signaling, and chemokine signaling), T cell receptor signaling, and Th1/Th2/Th17 cell differentiation, suggesting an immunosuppressive status in MLNs of CRC model. The DIA approach using the immune cell-enriched libraries showcased deep coverage and high sensitivity that can facilitate illumination of the immune proteomic landscape for microscale samples.

## Highlights

• A spectral library resource of 7815 proteins across six primary immune cell subtypes.
• Library-assisted DIA offers deep coverage for immune proteomics in microscale tissue.
• Library-assisted DIA enables sensitive mapping of immunological proteins in TME.
• Application in CRC mouse models revealed immunosuppression in mesenteric lymph nodes.

## Graphical Abstract

**Figure:**
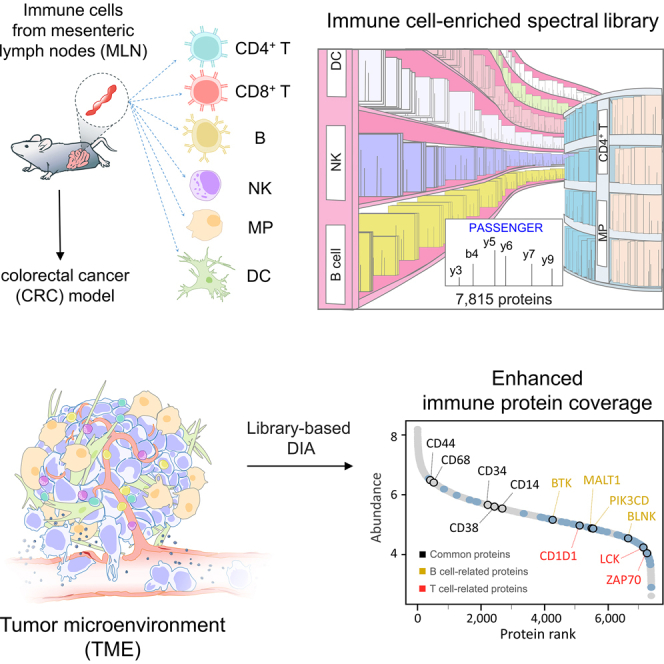

## In Brief

For highly sensitive immune cell profiling in the tumor microenvironment, six immune cell subtype-specific spectral libraries covering 7815 proteins were established as a community resource. The library-assisted single-shot data-independent acquisition strategy offers deep coverage and identification of low-abundance immunobiologic process-related proteins in microscale tissue (1 μg), providing comprehensive coverage of surface markers and key signaling pathways. Application in colorectal cancer mouse models revealed an immunosuppressive status in mesenteric lymph nodes, confirming the feasibility of the strategy for future clinical sample applicability.

The tumor microenvironment (TME) is a complex arena with varying compositions of immune cells, stromal cells, tumor vasculature, and the extracellular matrix. Tumor progression is determined by the dynamic and reciprocal relationship between cancer cells and the components of the TME, causing chaotic angiogenesis and alteration of the composition of the cells, extracellular matrix, and vascular patterns (1). Immune cells infiltrating the TME play critical roles in shaping tumor development with their functions of tumor-antagonizing or tumor-promoting, thus affecting chemotherapy and immunotherapy efficacy and prognosis (2, 3). Therefore, targeting immune system components, such as blockade antibodies for immune checkpoint modulators CTLA-4 and PD1 in T cells, and cluster of differentiation 40 (CD40) agonists for macrophage (MP) infiltration, have emerged as a promising strategy for tumor immunotherapy (4, 5, 6). Immune cell infiltration, recruited by inflammatory signals and stromal cells in the tumor core, margins, or sentinel lymph nodes (LNs), has a significant impact on cancer prognosis (7, 8, 9). Sentinel LNs, such as mesenteric lymph nodes (MLNs) in colorectal cancer (CRC), control B and T cell activation and may signal cancer cell invasion of the LNs (10). The activity of immune cells in MLNs is often suppressed by invading cancer cells, thereby facilitating the spread of cancer cells (11, 12). However, many mysteries remain, such as the uncertain mechanism behind LN yield that provides a survival advantage in CRC (13). During tumor growth, the TME accumulates immunosuppressive molecules that are secreted by cancer cells, allowing the cancer to evade immunosurveillance (14) and lead to resistance to therapy (15, 16). Understanding the changes in immune cell profile in TME or sentinel LNs during tumor growth has helped develop tailored therapeutic strategies. However, only a relatively low percentage (<15%) of patients respond to immunotherapy (17). It is critical to develop a profiling platform capable of resolving the immune heterogeneity of TME and temporal changes in the immune cell composition in sentinel LNs.

Advancement in mass spectrometry (MS)-based techniques has facilitated in-depth proteomics profiling of clinical samples, enabling phenotype classification, biomarker discovery, and nominating therapeutic opportunities toward precision oncology (18, 19, 20). Tissue biopsies are common clinical samples for diagnosis and prediction of immunotherapy prior to treatment (21), yet their in-depth proteomic analysis is often compromised by limited tissue amounts. The immune cells within TME represent small yet highly dynamic populations with varied proportions, depending on the tissue type and individual. Various proteomics studies have attempted to provide a systematic view of the molecular features among immune cell subtypes. One potential solution to analyze such low cell populations in the TME is to isolate each cell type at the price of large amounts of starting materials. For instance, Rieckmann *et al.* constructed a proteomic resource (ImmProt) of 28 hematopoietic cell types and identified over 10,000 proteins from millions of sorted immune cells of healthy human donors (22). Combining tandem mass tag labeling and stop-and-go extraction tip (StageTip) fractionation, Myers *et al.* established a profile of 12 immune cell types in mice, with coverage over 7000 proteins from a starting amount of 300,000 cells per cell type (23). A recent study used data-independent acquisition mass spectrometry (DIA-MS) to characterize fluorescence-activated cell sorting (FACS) purified immune cells (T cells, natural killer (NK), and MPs) isolated from 48 hepatocellular carcinoma patients, revealing the phenotype alterations of enriched THY-1 and SGPL1 in MPs and AFAP1L2 in T cells within the TME (24). In the above research, the in-depth immunoproteomic profiling was achieved at the expense of prior cell isolation, extended liquid chromatography-tandem mass spectrometry (LC-MS/MS) acquisition time, and large amounts of samples, making it challenging to apply to size-limited samples such as rare cell populations or minute clinical specimens. Highly sensitive approaches to allow deep profiling of the immune proteome in the TME are needed to illuminate the heterogeneity and interplay of the immune cell population in the TME.

Although the spleen and LNs have been extensively studied in the context of cancer immunity, little is known about the conversion of immune cell profiles between sentinel LNs and tumors during cancer progression. Furthermore, the proteomic alteration of immune cell infiltration in draining LNs that associate with metastatic outcome may provide insight to improve the immunotherapy toward personalized management. In this study, the MLNs in mice with CRC from orthotopic injection of murine colon 38 (MC38) cells were used as a model to develop an immune cell subtype library-assisted DIA-MS (libDIA-MS) strategy to enhance the immune cell profiling performance in the TME. Specifically, six immune cell subtype-specific libraries were constructed using microscale samples isolated from the major immune cells of MLNs, including CD8^+^ and CD4^+^ T lymphocytes, B lymphocytes, NK cells, dendritic cells (DCs), and MPs. These libraries comprised 7815 protein groups (97,477 precursors and 76,488 peptides), including 969 immunobiological process-related proteins annotated from the Gene Ontology (GO) database. The feasibility of single-shot DIA for microscale tissue profiling was evaluated by 1 μg of tumor from the murine CRC model. The immune cell-enriched libDIA significantly enhanced the sensitivity and coverage for various immune cell markers in the proteomic landscape in the CRC tumor. Finally, application on the MLNs of CRC models revealed the immunosuppressive status in the TME landscape, nominating a number of alternative protein markers and key immune pathways. These results demonstrated an approach for unraveling the complex immune proteome and immune heterogeneity in the microenvironment of minute tissues.

## Experimental Procedures

### Mouse Strains and Cell Lines

Seven-to eight-week-old C57B/6 mice (male) were purchased from the National Laboratory Animal Center. All animal experimental procedures were approved by the Institutional Animal Care and Utilization Committees (IACUC, 21-11-1729) of Academia Sinica and were carried out in accordance with relevant national and international guidelines. The murine CRC cell line MC38 (Cat. No. ENH201-FP) was purchased from Kerafast, Inc. Cells were grown in Dulbecco's Modified Eagle Medium (Cat. No. 11965092, Gibco, Thermo Fisher Scientific) supplemented with 10% fetal bovine serum (Cat. No. 26140079, Gibco) and penicillin (100 U/ml)/streptomycin (100 μg/ml) (Cat. No. 15140148, Gibco). Cells were incubated at 37 °C under an atmosphere containing 5% CO_2_.

### Experimental Design and Statistical Rationale

In this study, six immune cell-specific libraries, including CD8^+^ and CD4^+^ T cell, B cell, NK cell, DC, and MP, were generated to enhance immune cell profiling performance in the TME. Given the low cell numbers of immune cells and the diverse stages of immune cell differentiation within the TME, it is technically challenging to achieve high proteome coverage for each immune cell type using sorted immune cells from tumors. Alternatively, immune cells in MLNs provide better proteome coverage for the immune cell spectral libraries. Thus, 50,000 cells from each immune cell type were sorted from MLNs of mice to construct the immune cell spectral libraries. Cells were sorted in steady-state using a flow cytometer or magnetic beads. For deep profiling of the tissue microenvironment, libDIA was performed to analyze samples from the MC38 cell-inoculated colon tracts and the corresponding MLNs. To demonstrate the feasibility of the immune cell subtype-specific libraries, proteomic profiles of MLNs from CRC and control mice were compared. All tissues were prepared and analyzed in triplicates using single-shot DIA, with the inclusion of indexed retention time (iRT) peptides for retention time alignment. For DIA analysis, label-free quantification was conducted at the MS2 level. The total cycle was designed as one MS survey scan followed by 40 MS/MS scans that subdivide a mass range of 400−800 *m/z* into 40 isolation windows of equal width, making up a total cycle time of 3 s. Statistical comparisons of protein expression between CRC and normal controls were performed using the two-sided Welch’s *t* test (*p*-value < 0.05).

### Orthotopic Implantation

An intrarectal injection model for colon cancer was performed as previously described (25). For this experiment, we harvested the MC38 cells at approximately 80% confluence and generated cell suspensions (10^5^ cells in 10 μl PBS) that were injected into the rectal submucosa of anesthetized C57BL/6 mice. Tumor samples and MLNs were obtained from the MC38 recipients and control mice injected with PBS 7 days later. The percentage of lymphocytes (CD45^+^Villin^−^collagen III^−^) in colon tissue was quantified in a 0.5 cm section, including a site inoculated with MC38 cells. Each section was digested with collagenase (2 mg/ml; Cat. No. SKU 11088882001, Roche), and lymphocytes were detected with the monoclonal antibody (mAb) against CD45 (clone 30-F11, BD Biosciences), the mAb against Villin (clone SP145, Abcam), and the mAb against collagen III (clone EPR17673, Abcam). Villin and collagen III antibodies were labeled using a Lightning-link Fluorescein Labeling Kit (Cat. No. 707-0010, Innova Biosciences) and a Pacific Blue Antibody Labeling Kit (Cat. No. P30013, Thermo Fisher Scientific), respectively, according to the manufacturer’s instructions. Analysis of the expression pattern of immune cell markers in the MLNs of MC38 recipients and control mice was evaluated using the following antibodies: Anti-CD3e-APC mAb (clone 145-2C11, BD Biosciences). Anti-CD4-FITC mAb (clone RM4.5, BD Biosciences), anti-CD27-Brilliant Violet 421 (clone LG.3A10, BD Biosciences), and anti-CD95-PE-Cy7 mAb (clone Jo2, BD Biosciences).

### Immune Cell Enrichment

MPs (CD11b^+^Ly6G^–^F4/80^+^), DCs (CD11b^+^Ly6G^–^MHC II^+^CD11c^+^), and NK cells (CD3e^–^B220^–^NK1.1^+^) were sorted using the BD FACSAria II flow cytometer. Single-cell suspensions of lymphocytes from the MLNs were stained with the following antibodies: anti-CD11b-APC-Cy7 mAb (clone M1/70, BD Biosciences), anti-Ly6G-Alexa Fluor 647 mAb (clone 1A8, Biolegend), anti-MHC II-PE mAb (clone M5/114, BD Biosciences), anti-CD11c-FITC mAb (clone N410, Biolegend), anti-F4/80-brilliant violet 421 mAb (clone BM8, Biolegend), anti-B220-PE mAb (clone RA3-6B2, BD Biosciences), anti-CD3e-FITC mAb (clone 145-2C11, BD Biosciences), and anti-CD161/NK1.1-APC mAb (Cat. NO. FAB76141A, R&D Systems). The applied staining and sorting methods were conducted as described previously (26, 27). B cells were enriched by positive selection with mouse CD45R (B220) microbeads (Cat. NO. 130-049-501, Miltenyi Biotec), CD4^+^ T cells were enriched by negative selection with the mouse CD4^+^ T cell isolation kit (Cat. NO. 130-104-454, Miltenyi Biotec), and CD8^+^ T cells were enriched by negative selection with the mouse CD8^+^ T cell isolation kit (Cat. NO. 130-104-075, Miltenyi Biotec). To determine the purity of the samples enriched with the isolation kit, we stained the cell fraction with markers for T cells (CD4: clone RM4.5, BD Biosciences; CD8: clone 53-6.7, BD Biosciences) or B cells (B220: clone RA3-6B2, BD Biosciences) and performed flow cytometric analysis (FACSCanto II Flow Cytometer; BD Biosciences) to assess purity.

### Cell and Tissue Lysis and Protein Digestion

For processing samples of sorted cells, 0.5 ml LoBind tubes (Eppendorf) precoated with 0.01% n-Dodecyl-β-D-maltoside (Cat. No. 324355, Millipore) were used throughout the whole process to reduce sample loss due to surface adsorption. The sorted cells were washed in iced PBS three times, aliquoted to 50,000 cells per sample, and precipitated at 3000 rpm for 10 min. Then, cell pellets were lysed in a solution of 1% sodium deoxycholate (SDC), 10 mM tris(2-carboxyethyl)phosphine, 40 mM chloroacetamide, 100 mM Tris hydrochloride (pH 9) with Phosphatases Inhibitor Cocktail 2 (Cat#P5276, Sigma-Aldrich), and Phosphatase Inhibitor Cocktail 3 (Cat#P0044, Sigma-Aldrich). The mixtures were then heated at 95 °C and 1000 rpm in a shaking incubator for 10 min. The lysates were digested with LysC (1:20, w/w; FUJIFILM Wako) and trypsin (1:10, w/w; Promega) at 37 °C overnight with gentle shaking at 1000 rpm. The digestion was quenched with 0.5% trifluoroacetic acid, followed by a centrifugation step at 13,500 rpm for 10 min to collect the supernatant.

Tissues were prepared and analyzed in triplicates using single-shot DIA. Frozen tissues were thawed rapidly on ice, cut into small pieces, and washed with ice-cold PBS. One milligram of tissue was homogenized in 100 μl 1% sodium deoxycholate lysis buffer using a mechanical homogenizer (Precellys 24, Bertin Technologies). The residual debris was removed by centrifugation at 13,500 rpm for 10 min, and the supernatant was collected into a LoBind tube. The collected supernatants were heated at 95 °C with gentle vortexing at 1000 rpm for 10 min. After cooling down, the samples were sonicated at 4 °C for 5 min (high mode, 30s on, 30s off) with a Bioruptor Plus (Diagenode). The lysates were quantified using the BCA assay (Cat. No. 23235, Thermo Fisher Scientific), and 1 μg of each lysate was digested overnight with LysC (1:20, w/w; FUJIFILM Wako) and trypsin (1:10, w/w; Promega) at 37 °C with shaking at 1000 rpm. The resulting peptide mixtures were acidified using 0.5% trifluoroacetic acid followed by desalting using house-made SDB-XC StageTips. In brief, the SDB-XC StageTips were prepared by packing one disc of SDB-XC membrane (Empore) into a StageTip using a 16-gauge needle, conditioned with 80% acetonitrile (ACN), and equilibrium with 5% ACN. Following sample loading, peptides were washed with 5% ACN and subsequently eluted using 80% ACN. Finally, the peptides were dried in a SpeedVac centrifuge and stored at −20 °C.

### High pH Reversed-Phase Peptide Fractionation

To increase protein identification coverage, reversed-phase peptide fractionation was performed for tryptic peptides from sorted cells using our previously reported high-pH reversed-phase C18 StageTip fractionation method (28). The StageTips were prepared by inserting reversed-phase membranes styrene divinylbenzene resin modified with sulfonic acid group (SDB-RPS) Empore disks into Eppendorf 200 μl tips and packing C18-AQ beads (1.25 mg, 5 μm; Dr Maisch-GmbH) on top. After loading the peptides into the C18 StageTips, 30 μl of 200 mM ammonium formate (pH 10) was added to switch the acidity. Peptides were eluted into seven fractions using a stepwise gradient with increasing ACN percentage (4%, 7%, 11%, 14%, 18%, 22%, and 80%) prepared in 200 mM ammonium formate. StageTips were centrifuged at 1500*g* for 2 min to elution of peptides from each fraction. The eluted peptides were desalted using house-made SDB-XC StageTips, dried in a SpeedVac centrifuge, and stored at −20 °C until further analysis by LC-MS/MS.

### LC-MS/MS Analysis

All samples were analyzed using an LTQ Orbitrap Fusion Lumos Tribrid mass spectrometer (Thermo Fisher Scientific) coupled with an UltiMate 3000 RSLCnano system (Thermo Fisher Scientific). Tryptic peptides were resuspended in 0.1% formic acid, spiked with iRT peptides (Biognosys), and loaded onto a 25 cm nanoEase M/Z Peptide CSH C18 Column (75 μm inner diameter, 1.7 μm particles of 130 Å pore size, Waters). Peptides were eluted with a nonlinear 90-min gradient of 3 to 40% ACN in 0.1% formic acid (3–25% from 0 to 72 min, 25–40% from 72 to 73.5 min, 40–95% from 73.5 to 75.5 min, washout at 95% for 4 min, and reequilibration at 1% for 10 min) at a flow rate of 250 nl/min and a column temperature of 45 °C.

For construction of the spectral library, the data-dependent acquisition (DDA) mode was used to obtain spectra for peptides from sorted cells. MS survey scans were acquired in the mass range of 395 to 1125 *m/z*, at a resolution of 120,000 at *m/z* 200. The standardized automatic gain control (AGC) was set with a target value of 4e5 and a maximum injection time of 50 msec. MS2 analysis was performed by automatic isolation of the top N intense precursors and fragmented within a cycle time of 3 s, with an intensity threshold of 5e4 using higher-energy collision dissociation at a normalized collision energy of 30%. Isolation windows were 0.7 *m/z*, and the dynamic exclusion time was set to 20 s. MS2 scans were recorded at an MS resolution of 30,000 with a maximum injection time of 54 msec with a standardized AGC target value of 5e4.

For tissue samples, the DIA mode was applied to generate comprehensive profiles. In DIA mode, the MS survey scan range was set to 395–1125 *m/z*, at a resolution of 120,000, with a standardized AGC target value of 4e5 and a maximum injection time of 50 msec. Precursors in a mass range of 400 to 800 *m/z* were subdividing into 40 isolation windows of equal width, and each window was fragmented with higher-energy collision dissociation at a normalized collision energy of 30% sequentially. For MS2 scans, the scan range was set to 145–1450 *m/z*, with a resolution of 60,000, 100% normalized AGC target (5e4), and dynamic maximum injection time mode.

### Construction of Spectral Libraries

Proteome spectral libraries for each immune cell subtype were constructed individually using Spectronaut Pulsar (v17.2, Biognosys). Raw files were searched against the SwissProt murine database (version: 2021_02, 17,073 entries) with the inclusion of the iRT peptide sequences. Either the DDA or DIA raw files were searched in a default setting that allows a maximum of two missed cleavages for peptide, fixed modification of cysteine carbamidomethylation, and variable modification of methionine oxidation and N-terminal acetylation. The dynamic mass tolerance correction factor for both MS1 and MS2 levels was 1. Deep learning assisted iRT regression was used, and the regression was accepted at a minimum R^2^ of 0.8. Fragment ions were acquired with minimum *m/z* 200, maximum *m/z* 3000, and minimal relative intensity of 5%. The false discovery rate (FDR) was set to 1% at peptide-spectrum match, peptide and protein levels. In the library, only unique peptides with at least three fragments and up to six fragments were included.

### Protein Identification and Quantification

DDA database search was performed by Proteome Discoverer 2.5 (PD2.5, Thermo Fisher Scientific) (29). The DDA raw files from sorted immune cells were analyzed using the Sequest HT search engine against the SwissProt murine database (version: 2021_02, 17,073 entries). For peptide identification, a maximum of two missed cleavages and a minimum peptide length of six amino acids were allowed, with carbamidomethylation on cysteine set as a static modification, and oxidation of methionine and deamidation of asparagine and glutamine set as variable modifications. The precursor mass tolerance was set to 10 ppm, and the fragment mass tolerance was set to 0.02 Da. The precursor mass range was set at 350 to 5000 Da. Search results were validated using target-decoy strategy in percolator with 1% strict and 5% relaxed FDR. Unique and razor peptides were used for quantification. Precursor abundances were calculated by summing the abundances of connected peptide groups. The output protein.txt files were then imported into Python for subsequent data processing and analysis, including normalization, database comparisons, Pearson’s correlation and Welch’s *t* test.

LibDIA and library-free (directDIA; dirDIA) DIA data analysis was performed using Spectronaut (v17.2). The SwissProt murine database (version: 2021_02, 17,073 entries) with the inclusion of the iRT peptide sequences was used for protein identification and quantification. For libDIA protein identification, the DIA dataset from tissues was analyzed against multiple spectral libraries with the standard setting. In general, a dynamic extracted ion chromatogram retention time extraction window correction factor of 1 and local (nonlinear) regression were used for peak extraction and retention time calibration. The dynamic mass tolerance correction factor for both MS1 and MS2 levels was set to 1. The FDR was set to 1% at precursor and protein levels using a mutated decoy database in 0.1 fraction library size. Peptide quantification was obtained at the MS2 level with at least three fragments. Protein abundance was calculated by averaging the quantities of the top one to three unique peptides according to their cross-run quality.

### Data Analysis and Pathway Annotation

Data analysis and visualization were performed in Python (3.7.4) with packages: pandas (1.3.5), scipy (1.7.3), scikit-learn (1.0.1), seaborn (0.11.2) and matplotlib (3.5.1). Protein abundance was normalized by the highest density centering to the kernel density estimation of the protein intensities for each raw dataset.

For the comparative analysis between the mouse immune cell subtype libraries and the human single-cell type-specific transcriptomes, the gene list of each immune cell type from the Human Protein Atlas (HPA) (version 22.0) (https://www.proteinatlas.org) (30, 31) was converted into homologous gene lists using HomoloGene (build 68) (32). We calculated the overlap percentage of homologous genes between HPA and our dataset within each immune cell subtype to evaluate protein/transcript composition similarity between datasets. For further comparison between the mouse immune cell subtype libraries and the ImmProt database (which contains human hematopoietic immune cell subtype proteomes), similar approach was performed by transforming the protein list of each immune cell type from ImmProt (http://www.immprot.org) (22) into lists of homologous proteins using HomoloGene (build 68) (32).

To generate the network plot of all immune cell subtype-specific library proteins, the protein lists from the six cell types were compared. The relative abundance of CD markers within each cell type proteome was determined by averaging the normalized abundance from the replicates of each cell type. The protein abundances were then ranked in ascending order and divided by the number of proteins identified in the cell type to determine their relative protein abundance rank.

For the quantitative comparison of the immune cell subtypes, proteins were filtered by detection in all replicates of a specific cell type, and imputed missing values in other proteomes with zero. Differential protein expression in each cell type *versus* all the other proteomes was quantitatively compared using a one-sided Welch’s *t* test with a *p*-value < 0.05. To identify differentially expressed immune proteins between MLNs of the MC38 recipients and control mice, a two-sided Welch’s *t* test (*p*-value < 0.05) was performed to quantitatively compare the immune proteins annotated from the GO database. Pathway enrichment of the differentially expressed immune proteins was performed using the Kyoto Encyclopedia of Genes and Genomes (KEGG) immune pathway database (https://www.kegg.jp/kegg) (33) in STRING (http://string-db.org) (v12.0) (34).

## Results

### Experimental Design of Immune Cell Subtype Library-Assisted DIA-MS

**Figure 1:**
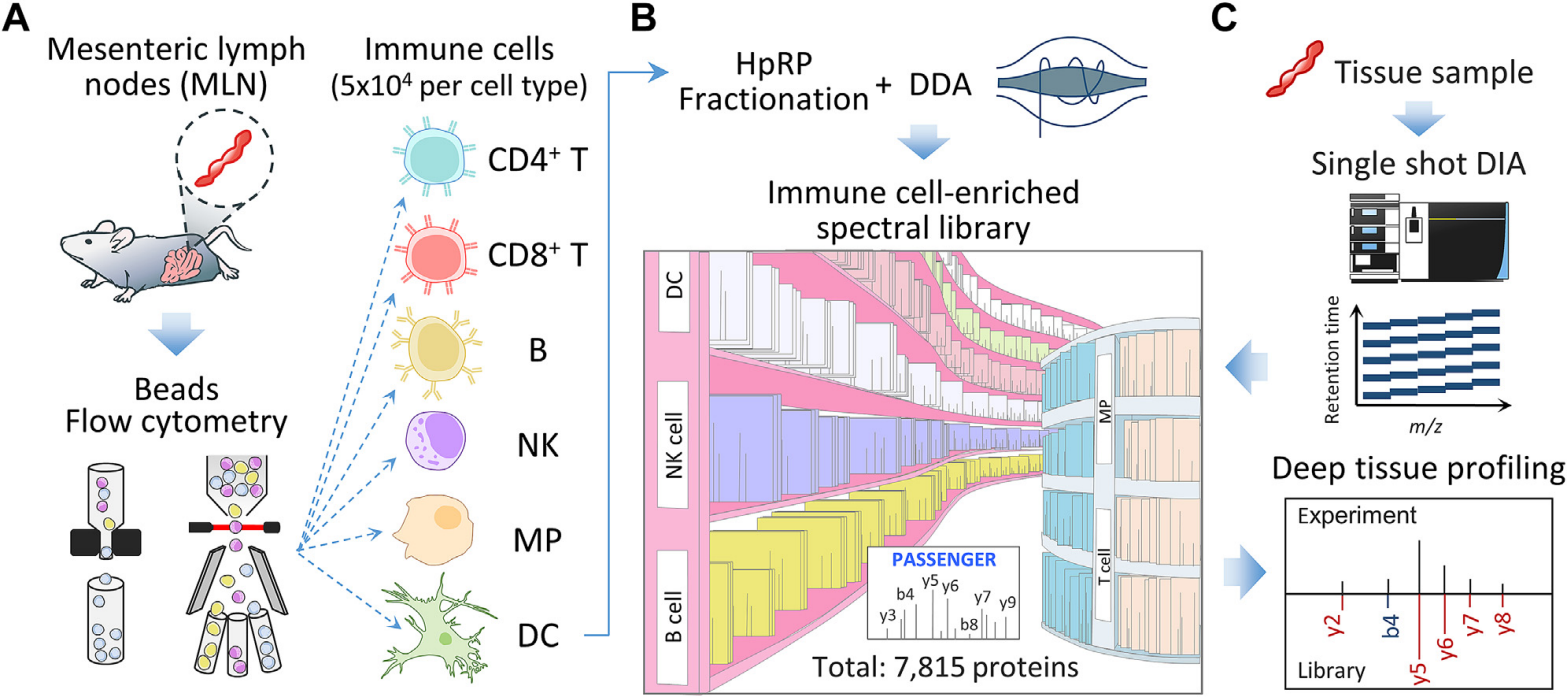
**Experimental design of immune cell-enriched libDIA.***A*, for construction of immune cell spectral libraries, CD8^+^ T cells, CD4^+^ T cells, B cells, NK, MP, and DC were isolated from MLNs using flow cytometry or magnetic beads. *B*, isolated cells were subjected to HpRP StageTips for peptide fractionation to increase the number of protein identification followed by DDA-MS analysis to generate six immune cell subtype-specific spectral libraries, which collectively constituted an integrated immune cell-enriched library of 7815 proteins. *C*, for enhanced sensitivity of immunoproteomic profiling at microscale amount, tissue samples were analyzed by single-shot DIA LC-MS/MS, followed by protein identification using the constructed immune cell-enriched spectral library. CD, cluster of differentiation; DC, dendritic cell; DDA, data-dependent acquisition; HpRP, high-pH reversed-phase; LC-MS/MS, liquid chromatography-tandem mass spectrometry; libDIA, library-assisted DIA; MLN, mesenteric lymph node; MP, macrophage; NK, natural killer; StageTip, stop-and-go extraction tip.

High-quality and deep coverage of reference spectral libraries are crucial for library-based DIA toward comprehensive proteomic profiling. Toward comprehensive coverage of reference mass spectra for immunoproteomics DIA analysis, we generated six immune cell subtype-specific spectral libraries using sorted immune cells from the MLNs of mice (Fig. 1*A*). These libraries comprised the major immune cell subtypes, including CD8^+^ T lymphocytes, CD4^+^ T lymphocytes, B lymphocytes, NK cells, DCs, and MPs. For each immune cell type, ∼50,000 cells were sorted using a cell sorter or microbeads. To increase the number of protein identifications for each immune cell type, the digested peptides from sorted cells were subjected to high-pH reversed-phase StageTips for peptide fractionation, followed by DDA-MS analysis (Fig. 1*B*). With the combination of the six immune cell-specific datasets, an immune cell-enriched spectral library resource consisting of 7815 protein groups was established, which was incorporated into conventional library-based DIA analysis to enhance the identification coverage of low-abundance immunobiological process-related proteins. To explore the effectiveness of our strategy in enhancing proteomic coverage of immune cells associated with CRC, a mouse model was established by orthotopic implantation of MC38 tumor cells (25). Colon tissues from the MC38 mouse model were analyzed by single-shot DIA LC-MS/MS, followed by the immune cell-enriched libDIA computation to evaluate enhanced sensitivity (Fig. 1*C*). Finally, the approach was used to quantitatively compare tissue proteome profiles of MLNs between MC38 and control mice to characterize cancer-related immunological alterations.

### Construction of Immune Cell Subtype-Specific Spectral Libraries

**Figure 2:**
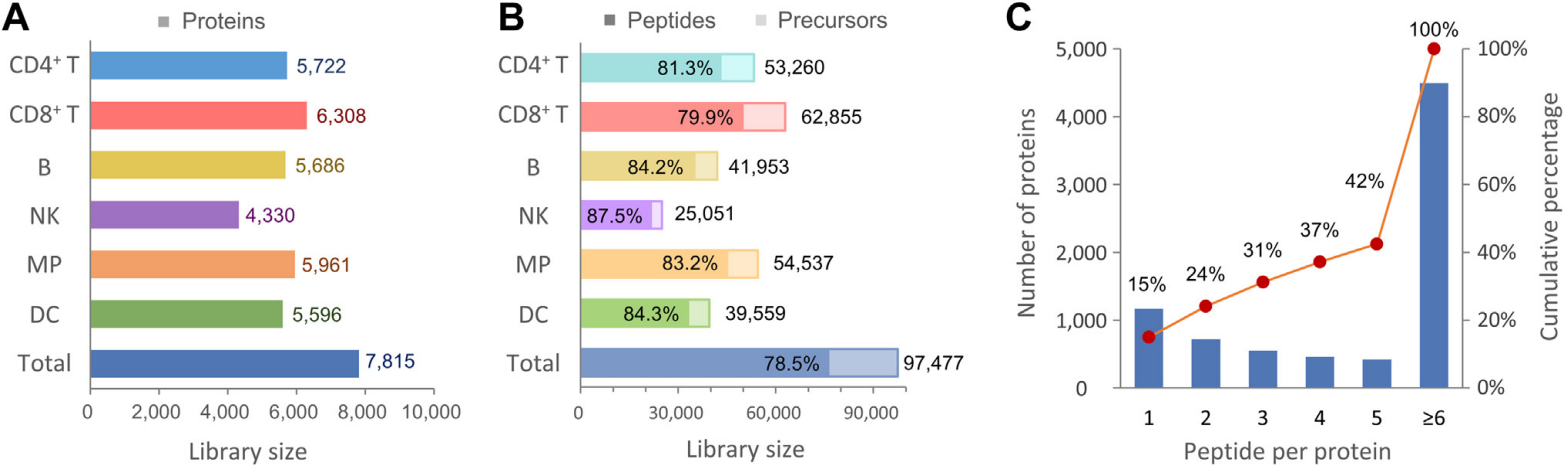
**Coverage of the immune cell subtype-specific spectral libraries.** Summary of six immune cell spectral libraries constructed from CD8^+^ T cells, CD4^+^ T cells, B cells, NKs, MPs, and DCs isolated from MLNs in CRC mice. Comparison of number of (*A*) proteins and (*B*) corresponding unique peptides and precursors in each immune cell subtype-specific library. *C*, distribution of the number of unique peptides per protein in all the libraries. The libraries contain an average of 4.5 unique peptides per protein. The *orange line* depicts the cumulative percentage of protein with six or more peptides. CD, cluster of differentiation; CRC, colorectal cancer; DC, dendritic cell; MLN, mesenteric lymph node; MP, macrophage; NK, natural killer.

For each immune cell type, ∼50,000 cells of sorted cell subtypes were lysed and subjected to protein digestion using trypsin. To confirm cell purity and success of the cell isolation procedure (supplemental Fig. S1, *A* and *B*), sorting purity by cell sorter (for DCs, MPs, and NKs), or by microbeads (for CD4^+^ T, CD8^+^ T, and B cells) was determined by flow cytometric analysis (supplemental Fig. S1, *A* and *B*, respectively). To maximize the enhanced identification coverage, the protein digests were subjected to high-pH reversed-phase fractionation followed by DDA-MS. It is noted that the spectral library was constructed using low cell numbers that are comparable to the amount of microscale samples, *i.e.*, μg of tissue. Thus, immune cell-enriched libDIA may achieve optimal sensitivity to increase identification coverage for microscale samples (35). Figure 2, *A* and *B* show a summary of identified proteins, peptides, and precursors in the six immune cell-specific spectral libraries (supplemental Table S1). At an FDR of 1% at the peptide-spectrum match, peptide, and protein levels, the CD8^+^ T and CD4^+^ T cell libraries were constructed with a total of 50,195 and 43,311 unique peptides corresponding to 6308 and 5722 protein groups, respectively. By a similar approach, the B cell and DC libraries comprised 35,312 and 33,364 unique peptides, which mapped to 5686 and 5596 proteins, respectively. The MP library comprised 45,398 peptides and 5961 proteins, while the NK library contained 21,924 peptides and 4330 proteins. Combining all the datasets (n = 112 raw files), the six immune cell subtype libraries constituted an immune cell-enriched library with a total of 76,488 peptides from 97,477 precursors, corresponding to 7815 protein groups.

Peptide detectability, defined as the probability that a peptide is identified in an LC-MS/MS experiment, is considered an important feature to observe a peptide from the sample (36). We next evaluated the quality and spectral features of the libraries, including number of peptides assigned to each protein, proteotypic peptide length and miscleavages of peptides (37). To improve identification of low-abundance proteins, which usually generate less comprehensive fragmentation patterns, without sacrificing specificity (35), the top three to six most intense fragment ions were retained for each precursor ion in each spectral library. The overall results correspond to an average of 4.5 unique peptides per protein in the library. Among them, the majority (58%, 4496 proteins) of the immune cell proteome was represented by five or more unique peptides per protein (Fig. 2*C*). In the integrated immune cell-enriched library, the majority of the peptides (93.1%) contains no missed cleavage, with only 6.7% and 0.3% having one and two missed cleavages, respectively (supplemental Fig. S2*A*). Additionally, 85% (6648 proteins) of the immune cell proteins from the library were represented by at least two quantifiable peptides per protein, meeting the criteria for unambiguous protein quantification (38, 39). Furthermore, the peptide lengths varied from 7 to 50 amino acids, with over 90% falling within the range of 7 to 20 amino acids, ensuring the good detectability in LC-MS/MS (supplemental Fig. S2*B*). These results demonstrate the overall good quality of the immune cell proteins-derived peptides within the spectral library.

### Characteristic of Immune Cell-Specific Proteome Libraries

**Figure 3:**
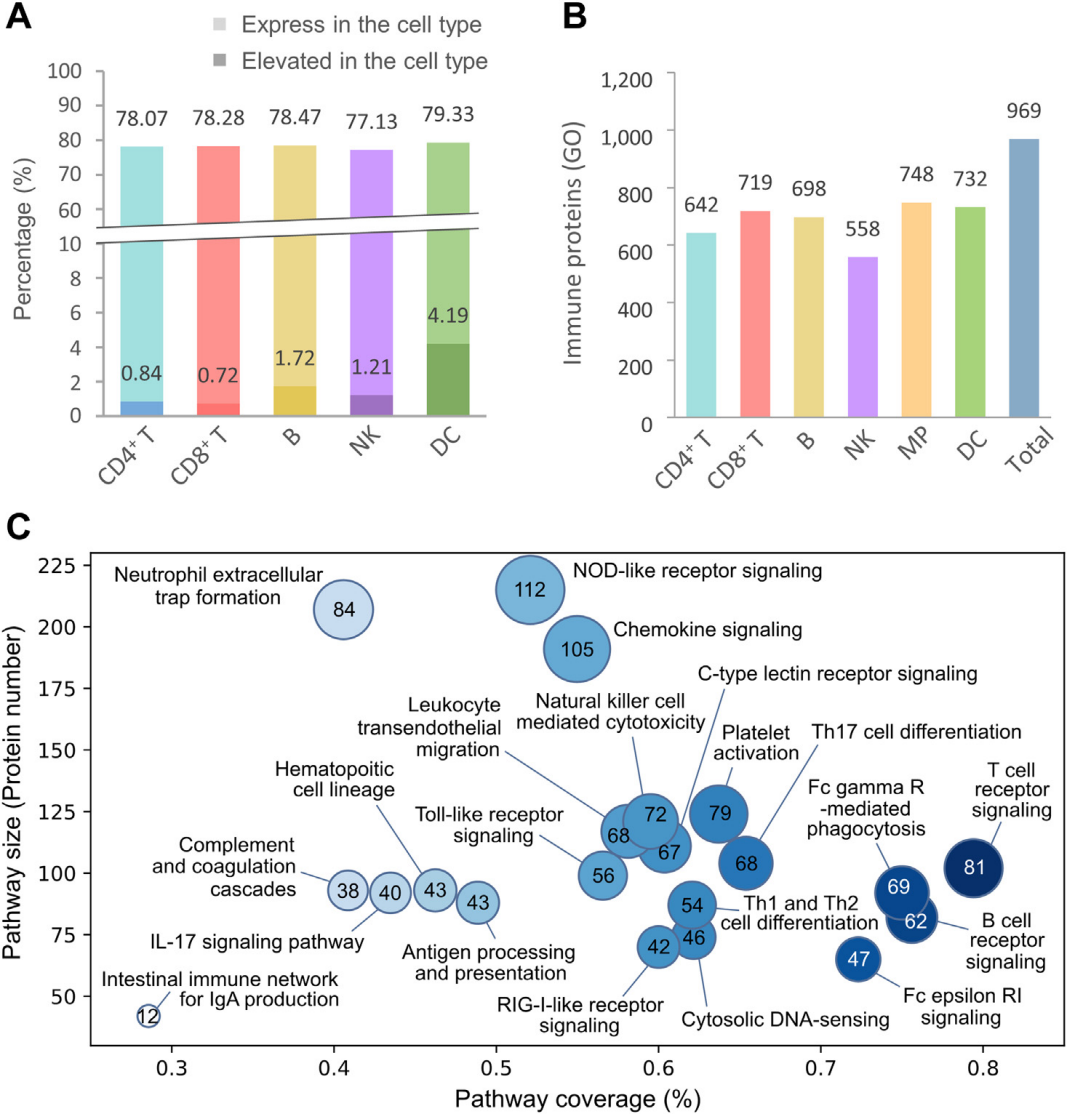
**Composition and characteristics of the immune cell subtype-specific spectral library.***A*, percentage of the coverage of homologs in the immune cell subtype-specific libraries among the corresponding human single-cell transcriptome in the HPA. The *darker color* indicates the percentage of overexpressed protein in each cell type. *B*, annotation by GO reveals numbers of immune function-related proteins in our library. *C*, enrichment analysis by KEGG database shows immune pathways in each library. The size of the *circle* corresponds to the protein coverage, while the X and Y axes depict protein coverage within the pathway and the pathway sizes, respectively. GO, Gene Ontology; HPA, Human Protein Atlas; KEGG, Kyoto Encyclopedia of Genes and Genomes.

Each immune cell type may possess proteome specificity characterized by its differential expression patterns of proteins compared to the general expression across all cell types (40, 41). To evaluate the proteome specificity of our immune cell subtype libraries, we compared our dataset with the single-cell transcriptome data of immune cells from the HPA (30, 31). We assessed the consistency in the protein composition between each immune cell subtype proteome library and the human transcriptome of the corresponding cell type, including CD4^+^ T, CD8^+^ T cells, B cells, NKs, and DCs, from the HPA (30, 31). The comparison was performed by converting the human genome-wide RNA expression profiles of single-cell immune cell types into mouse homologs using the HomoloGene database provided by the National Center for Biotechnology Information (32). Then we calculated the percentage coverage of the human cell subtype homologs among its corresponding mouse immune cell subtype-specific library. On average, 78.26% of the proteome of each immune cell subtype was covered by the single-cell transcriptome of the corresponding human cell subtype (Fig. 3*A*). We also filtered the proteins that showed elevated RNA expression among each immune cell type, which likely present the immune cell-specific markers. The analysis reveals 4.19% (233 proteins) of the DC proteome mapped to transcript with elevated RNA levels compared to other cell types, suggesting potential DC-specific upregulated proteins (Fig. 3*A*). Likewise, a small percentage (1%) from other immune cell-specific proteomes, including CD4^+^ T cell (48 proteins), CD8^+^ T cell (45 proteins), B cell (97 proteins) and NK cell (52 proteins), also matched to the transcripts with elevated expression at the transcriptomics level compared to other cells. Despite the differences in organism-specific proteins between our mouse datasets and the HPA, these results suggest that our immune cell subtype proteomes had high agreement with previously reported human transcriptomes and show immune cell-specific protein subsets.

To further align the organism specificity (mouse-human) for an unbiased comparison of the specificity of the proteomes from different immune cell types, we compared our immune cell subtype libraries with the human hematopoietic immune cell proteomes from the ImmProt database, which dataset was established by DDA analysis from large-scale sorted cells (supplemental Fig. S3) (22). For species conversion between human and mouse, each human immune cell subtype proteome from the ImmProt database was compared to the corresponding mouse homologs using the HomoloGene database (32); the number of mapped homologs varied in different cell types. Specifically, in the steady-state NK and DC datasets, 8165 and 8289 proteins had matched mouse homologs in the HomoloGene database, respectively. However, relatively lower numbers of homologs were matched for the CD8^+^ T (7802), CD4^+^ T (7498), and B cell (7362) datasets (supplemental Fig. S3). By overlapping the above-mentioned human homologs with our mouse proteome from the established immune cell libraries, an average of 80% of each immune cell subtypes-specific library was covered by the human immune cell subtype proteome (supplemental Fig. S3), demonstrating the strong consistency between our immune cell subtype-specific libraries with the human immune cell proteomes from the ImmProt. Apart from the common proteins, our libraries exclusively cover immunological markers such as CD1D1 in the CD4^+^ T cell and NK cell-specific libraries, and CD209 in the DC-specific library. It is noted that both CD markers were not present in the ImmProt database though they have human homologs. The results suggest the specificity and complementarity of our library and improved sensitivity by using significantly less amount of immune cell input in this study.

Finally, we evaluated the coverage of immune function-related proteins in our library. GO category analysis revealed that a total of 969 proteins in our library are annotated to be involved in the biological process of the immune system. Among the individual cell type datasets, MPs, DCs, CD8^+^ T cells, and B cells exhibited coverage of about 700 immunobiological process-related proteins, while CD4^+^ T cells and NK cells showed slightly lower coverage of 642 and 558 immunobiological process-related proteins, respectively (Fig. 3*B*). These immunobiological process-related proteins were also annotated using the KEGG database (Fig. 3*C*) (33). A total of 604 protein groups were annotated as involving in immune system pathways with coverage ranging from 29% to 79%. Key immune pathways were mapped with high coverage, including T cell receptor signaling (81 proteins, 79%), B cell receptor signaling (62 proteins, 76%), and Fc gamma receptor-mediated phagocytosis (69 proteins, 75%). Additionally, the immune cell-enriched library also shows substantial overlap with pathways involved in CD4^+^ T cell development and lineage differentiation, such as Th1 and Th2 cell differentiation (54 proteins, 62%) and Th17 cell differentiation (68 proteins, 65%), and pathways related to immune cell effector functions such as platelet activation (79 proteins, 64%) and NK cell-mediated cytotoxicity (72 proteins, 60%). These results indicate that the established immune cell-enriched spectral libraries have high coverages of immune function-related proteins and pathways for immunoproteomics analysis.

### Evaluation of Immune Cell Protein Markers and Subtype-Specific Proteome

**Figure 4:**
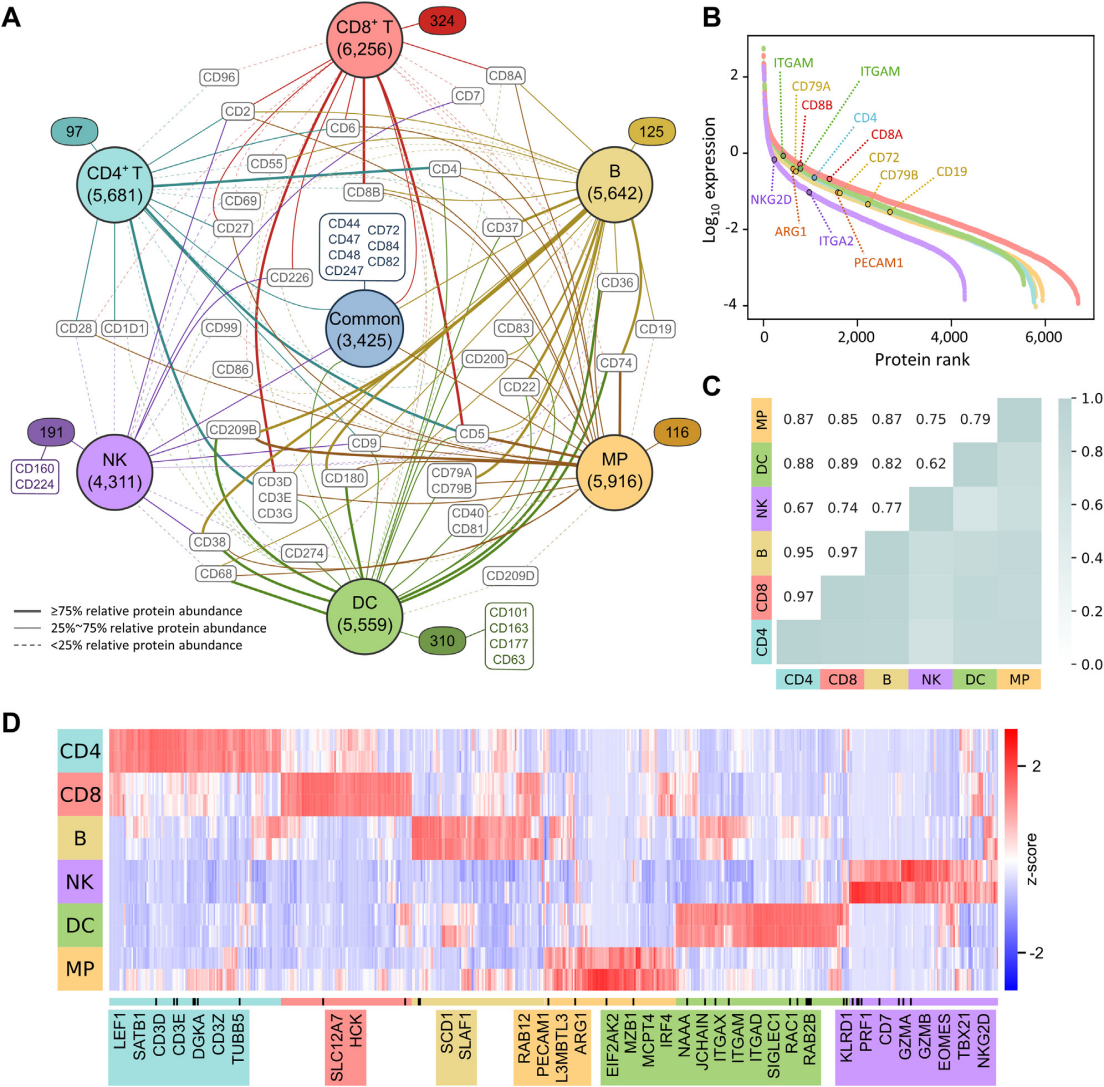
**Evaluation of immune cell subtype specificity and protein markers.***A*, the network plot visualizes the overlap of CD markers in the immune cell type-specific libraries. *Connected lines* indicate the presence of CD markers within the subtype library. *Thick lines* denote CD markers with a relative abundance higher than 75% within their respective cell type proteomes, confirming their high specificity among immune cells. The *dash lines* signify trace expression (<25% relative protein abundance) within corresponding cell type. *B*, relative protein abundance plot of immune cell subtype proteomes and selected immunophenotypic markers. *C*, pairwise Pearson’s correlation analysis of the protein expression within immune cell subtype proteomes. *D*, heatmap of the top 100 significantly DEPs of each cell subtype (CD4^+^ T cell, CD8^+^ T cell, B cell, NK cell, DC, and MP; one-tailed Welch’s *t* test, *p* < 0.05). Columns were clustered with Euclidean distance and rows were ordered by cell lineage as shown in the legend. Selected examples of protein names are shown in the indicated clusters. CD, cluster of differentiation; DC, dendritic cell; DEP, differentially expressed protein; MP, macrophage; NK, natural killer.

To evaluate protein specificity across different immune cell type-specific proteomes, we compared the proteome composition of the six immune cell types. Additionally, we quantitatively evaluated the abundance of CD markers present across each cell type. Subsequently, a network plot of all immune cell subtype-specific library proteins was constructed to illustrate the connectivity between these previously annotated CD markers and each cell subtype library. It is noted that different widths of lines connecting CD markers to immune cell subtype libraries indicate their relative abundance in the ascending ranking within each cell subtype proteomes (Fig. 4*A*). The network plot revealed that only half of the total proteins (3425 proteins) were commonly identified in all the cell subtypes, while >1000 proteins were exclusively identified in one specific cell subtype library. Furthermore, a total of 50 CD markers were identified across all the cell subtypes, distributed as follows: 26 in CD4^+^ T cells, 27 in CD8^+^ T cells, 37 in B cells, 24 in NK cells, 39 in MPs, and 40 in DCs. Among these, six CD markers were exclusively expressed within a specific immune cell subtype library, with four in DCs and two in NKs. The subtype-specific proteins include CD markers such as CD244 that activates the cytotoxicity and interferon-γ production in NK cells and the scavenging receptor CD163 in DCs (42, 43). The majority of CD markers, 44 in total, were shared with differential abundances across at least two libraries. Though showing trace expression (<25% relative protein abundance) in other cell types, these subtype-specific markers are among the high abundance range (>75%) within their respective cell type proteomes, confirming their high specificity among immune cells. Examples of such markers include CD3 complexes (CD3D, CD3E, and CD3G), which have trace abundance in the B cells, MPs, and DCs, yet show high expression levels (75–82%) in CD4^+^ T and (82–88%) CD8^+^ T cells. Other examples include high expression of CD4 in CD4^+^ T cells, CD37 and CD19 in B cells, and CD8A and CD8B in CD8^+^ T cells. The observed differences in the cell subtype-associated protein markers highlight the specificity of our immune cell subtype-specific libraries.

To assess whether the measured protein abundance recapitulates the expression patterns of immune cell types, we ranked the protein abundance for each cell subtype (Fig. 4*B*). Many immunophenotypic markers specific to each cell type were found exclusively abundant, exemplified by the known CD8^+^ T cell surface marker CD8B and CD8A in the higher ranked abundance of the CD8^+^ T cell proteome. Similar trends were observed in the marked abundances of CD4 in CD4^+^ T cell proteome and the medium-to-high abundance of CD79A, CD72, CD79B, and CD19 in B cell proteome. The data suggested that these canonical markers had higher expression in the corresponding cell types. With the presence of these canonical markers in our immune cell-enriched library, it is expected that the libDIA would provide good sensitivity for proteomic profiling of immune cells. To evaluate the similarity of the proteomes between individual cell subtypes, correlation analysis was performed across the different cell types using the mean protein expression of each replicates (Fig. 4*C* and supplemental Table S2). As expected, the correlation between T lymphocytes (CD4^+^ T and CD8^+^ T cells) was especially strong (r = 0.97). High correlations were also observed between T lymphocytes and B lymphocytes (r = 0.97 for B and CD8^+^ T cells; r = 0.95 for B and CD4^+^ T cells). On the other hand, the T and B cells had much lower correlation with the profiles of MPs and DCs (r = 0.82–0.89), while NK cells had a slightly weaker correlation with the other cell types. In general, the correlations between the individual cell subtypes agreed with the established cell lineage in immunology at the protein level, demonstrating the fidelity of the cell subtype proteomes.

To identify proteins that better distinguish different cell types, we performed one-sided Welch’s *t* test for differential protein expression in each cell type *versus* all the other proteomes. We filtered the proteins detected in all the replicates for a specific cell type to confirm its reproducibility and imputed missing values in other datasets with zero. The top 100 significantly differential proteins for each cell subtype were shown in the heatmap in Figure 4*D*. The differentially expressed proteins agreed with known lineage-specific markers. Expression of CD markers such as CD3E, CD3D, and CD3Z (CD247) for T cells, KLRD1 (CD94) for NK cells, and ITGAM (CD11B) and ITGAX (CD11C) for DCs was highly specific to the cell type. Apart from that, developmental stage-specific markers, such as EOMES and NKG2D (CD314), show expression signatures for NK cells (44). These expression patterns could provide alternative or additional markers for the immune cells. Taken together, these data indicate our immune cell subtype library sources recapitulate many known aspects of immunology and may provide a valuable source for immunoproteomics analysis.

### Immune Cell Subtype-Specific Libraries Enhanced Coverage of Immunoproteomic Landscape

**Figure 5:**
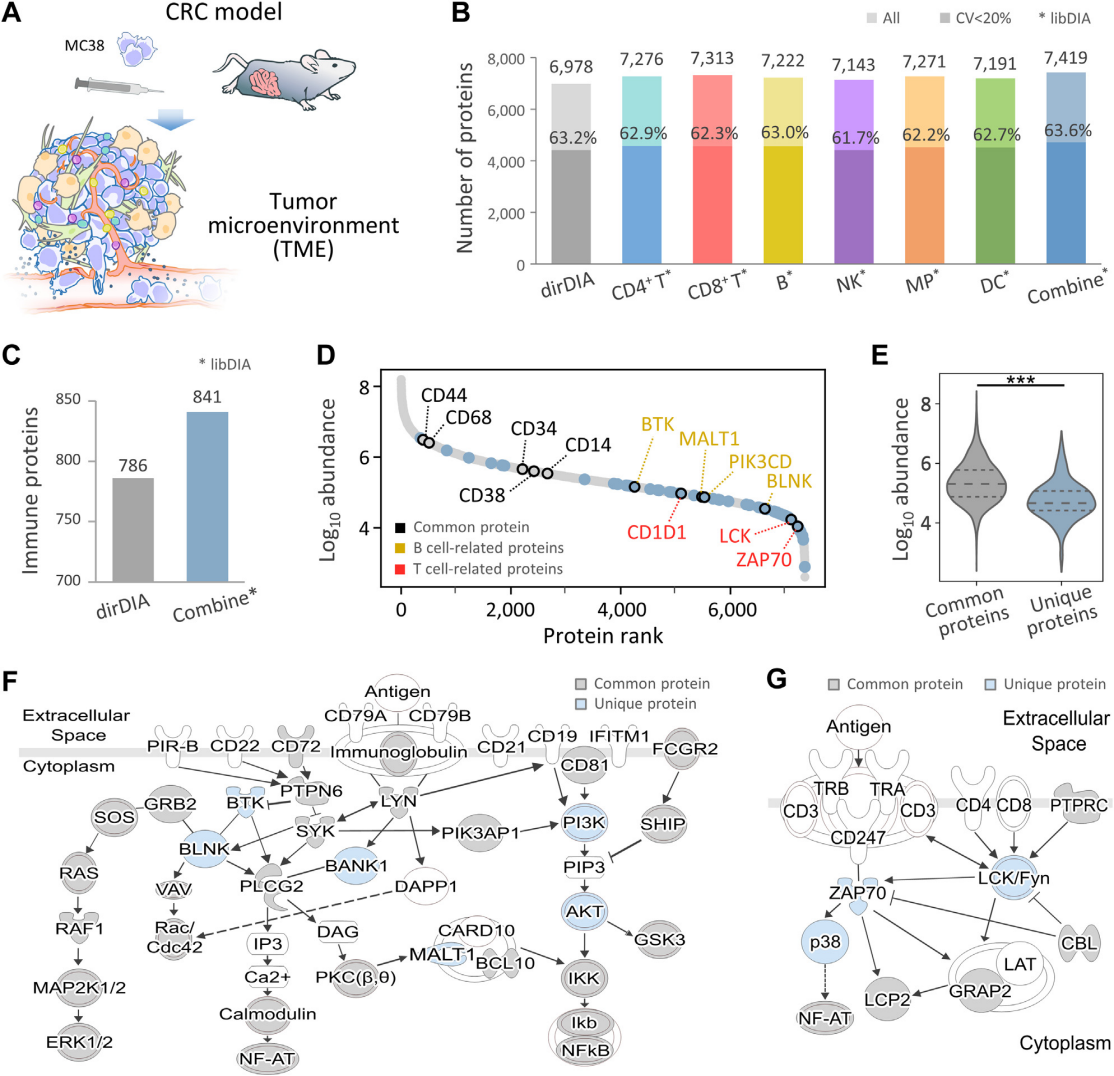
**Application of immune cell-enriched libDIA on 1 μg tissue protein from colon tissue of CRC mice.***A*, establishment of the CRC mouse model orthotopically inoculated with the CRC cell line (MC38 cells), providing a model for TME tissue for the benchmarking analysis. *B*, comparison of the number of proteins identified (*light color*) and quantified with coefficient of variation <20% (*dark color*) between dirDIA and immune cell libDIA using different immune cell-specific libraries. The combined library represents the integrated library of the six immune cell subtype-specific libraries. *C*, number of immune proteins identified using dirDIA and libDIA using the combined library. *D*, the relative abundance plot of the commonly identified proteins (*gray*) and exclusively identified immune proteins using libDIA (*blue*). Selected examples of commonly identified proteins (*black*), exclusively identified T cell-related proteins (*red*), and exclusively identified B cell-related proteins (*yellow*) are shown. *E*, comparison of the protein abundance distributions between the commonly identified proteins and exclusively identified immune proteins using libDIA (two-tailed Welch’s *t* test, *p* < 0.001). The three *dash lines* represent the interquartile range (IQR), with the median depicted by a line in the center. The whisker extends from 1.5 times the IQR quartile values of the data. *F*, enhanced protein coverage in the B cell receptor signaling pathway. *G*, enhanced protein coverage in the T cell receptor signaling pathway. *Gray nodes* depict commonly identified proteins, and *blue* nodes depict the exclusively identified proteins using libDIA. CRC, colorectal cancer; dirDIA, direct DIA; libDIA, library-assisted DIA; MC38, murine colon 38; TME, tumor microenvironment.

To benchmark the performance of immune cell-enriched libDIA, we applied single-shot DIA-MS for proteomic profiling in colon tumor tissue from mice orthotopically inoculated with MC38 cells, a CRC cell line derived from the C57/BL6 mouse. We injected 1 × 10^5^ MC38 cells into the rectal submucosa of anesthetized C57BL/6 mice to establish a mouse model of CRC (Fig. 5*A*). The tumor tissue samples were collected 7 days after implantation with an average size of 25 mm^3^ or less (supplemental Fig. S4*A*) which obtains a relatively low immune infiltration (supplemental Fig. S4*B*) (45). In the colon tracts of MC38 orthotopically inoculated sites in C57BL/6 mice, the percentage of CD45^+^ cells ranged from 0.25% to 3% (supplemental Fig. S6*B*), suggesting that the degree of immune cell infiltration in our samples is lower than in the immunosuppressed state (46).

To evaluate the feasibility for microscale tissue proteins, only 1 μg protein after tissue homogenization was used for proteomic processing to demonstrate the sensitivity of the developed workflow. For identification and quantitation, the dataset was processed with our libDIA and compared to the result from dirDIA analysis. By conventional dirDIA, a total of 6978 protein groups were identified from the colonic tissue (supplemental Table S3). To proceed with libDIA, there is flexibility to use either a single immune cell subtype-specific library or a combination of multiple cell subtype libraries depending on the study object. By using a specific cell subtype library of choice, the sensitivity and the coverage of the corresponding immune cell profile were enhanced. For example, by using CD4^+^ T cell library (size: 5722 proteins), additional 298 proteins were identified, increasing the coverage to 7276 proteins (Fig. 5*B*). This led to the exclusive identification of markers such as CD1D1 and CD2 (supplemental Fig. S5). Similarly, by using NK cell libDIA, an extra 165 proteins were identified, expanding the coverage to 7143 proteins and facilitating the exclusive identification of CD244, an immunoregulatory receptor that displays inhibitory signals in tumor-associated immune cells (supplemental Fig. S6) (47). On average, about 250 additional proteins were identified using immune cell subtype-specific libDIA of each single cell type. Finally, the combination of the six immune cell libraries yielded the coverage of 7419 proteins with an increase of 441 proteins compared to dirDIA, while the number of proteins quantified with coefficient of variation <20% increased by 307 (supplemental Table S4). The overlap between identified proteins using libDIA and dirDIA indicated that libDIA greatly enhanced the immune cell proteome coverage; almost all the proteins (99%) identified by dirDIA were covered by the immune cell-enriched libDIA (supplemental Fig. S7). Compared to the dirDIA approach, these results confirmed that the improvement achieved through the use of immune cell-enriched library-assisted approach is not merely due to the differences in data deconvolution, but mainly contributed from increased sensitivity gained from the immune proteome library with higher coverage than the identified proteins in the tissue samples.

By mapping the GO database, the immune cell-enriched libDIA identified more immunobiological process-related proteins (841) compared to the 786 immune proteins using dirDIA method (Fig. 5*C*). By rank-ordering the measured abundance of immune proteins commonly identified in both methods (gray in Fig. 5*D*) and additional proteins identified using the immune cell-enriched library (blue in Fig. 5*D*), many exclusively identified immune proteins were found positioned in the lower or lower-middle of the ranked abundance. Examples of these proteins include ZAP70, lymphocyte-specific protein tyrosine kinase (LCK), and CD1D1 that are related to T cells, and BLNK, MALT1, and BTK that are within the B cell receptor signaling pathway. A two-sided Welch’s *t* test revealed that the abundances of the exclusively identified immune proteins were significantly lower compared to the commonly identified proteins (*p*-value < 0.001) (Fig. 5*E*), highlighting the higher sensitivity of libDIA to identify the immune proteins present in the colon tract tissues with lower abundance, likely due to the trace amount of immune cells in the colon tract. To gain insight into the enhanced immune pathway coverage with the use of our approach, we mapped the proteomics profile of the CRC tumor onto the KEGG pathways of the immune system and cancer. In the example of B cell receptor signaling pathway, the key molecules such as Food and Drug Administration-approved druggable targets, PI3K and AKT, were exclusively identified by libDIA, increasing the coverage of PI3K-AKT signaling (Fig. 5*F*). The identifications of BTK and BLNK using our method also showed enhanced coverage of BCR-PLCG-Calcineurin signaling. Another example is the higher coverage of the T cell receptor signaling pathway, with the additional identification of LCK, ZAP70, and p38 mitogen-activated protein kinases (Fig. 5*G*).

In summary, these results demonstrate that immune cell-enriched libDIA offers deep coverage of the immune landscape, especially providing higher sensitivity for the detection of low-abundance immune proteins. By including diverse cell type-specific libraries in the libDIA analysis, more immune proteins can be identified toward comprehensive coverage of the immune cell proteomic profiles. With the demonstrated result from 1 μg tissue protein, the method has the potential to provide deep immune proteomic profiling for microscale tissue samples.

### Proteomic Profiles Reveal Evidence for Altered Immune Profiling in MLNs from Mice with CRC

**Figure 6:**
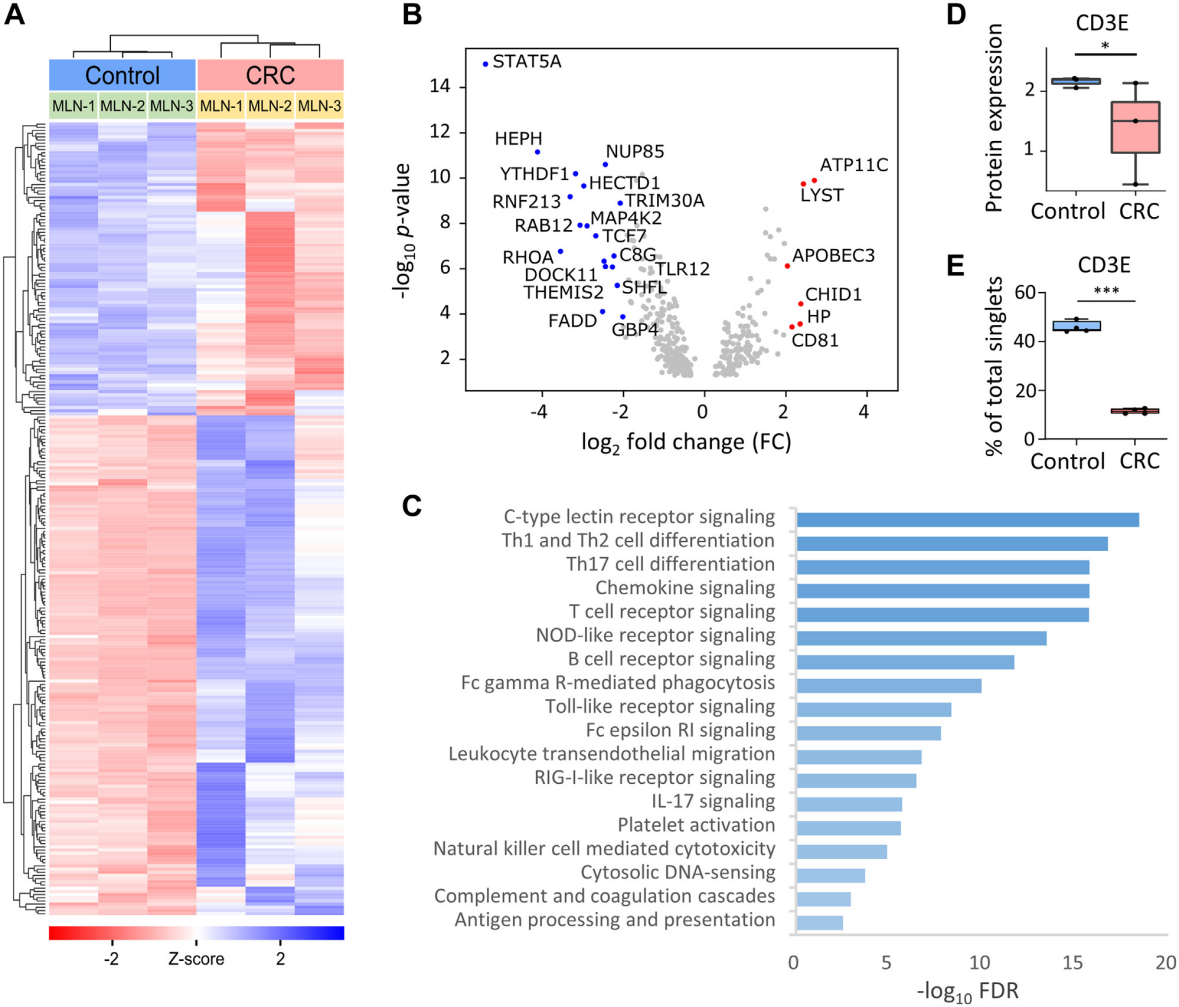
**Summary of differential immune proteome of MLNs from the MC38 cell-implanted CRC mouse model.***A*, unsupervised clustering of 348 differentially expressed immunobiological process-related proteins between MLNs of MC38-implanted CRC and control mice (two-sided Welch’s *t* test, *p* < 0.05). *B*, volcano plot of immunological proteins differentially expressed (two-sided Welch’s *t* test, *p* < 0.05) between MLNs of MC38-implanted CRC and control mice from three biological replicates. *C*, pathway enrichment of downregulated immunobiological process-related proteins using the KEGG immune pathway database (FDR<0.01). *D*, box plot displays the downregulated expression of CD3E in CRC mice using DIA-MS. *E*, flow cytometric characterization of CD3E in the MLNs of control and MC38 recipients. *Boxes* represent the interquartile range (IQR), with the median depicted by lines within the boxes. The *whisker* extends from 1.5 times the IQR quartile values of the data. CD, cluster of differentiation; CRC, colorectal cancer; FDR, false discovery rate; KEGG, Kyoto Encyclopedia of Genes and Genomes; MC38, murine colon 38; MLN, mesenteric lymph node.

Encouraged by the enhancement in proteome coverage of the immune landscape of colon tissue present in the CRC mice for microscale samples, we applied our immune cell-enriched libDIA approach to investigate the proteomics profiles of the MLNs of the CRC model. To explore the proteomic alteration of the immune microenvironment in MLNs and the underlying association between the reactive hyperplasia of sentinel LNs and tumor-infiltrating lymphocytes (TILs) in CRC, we quantitatively compared the proteomic profiles of the MLNs (1 μg tissue protein) from both MC38 implanted CRC model and control mice with three biological and three technical replicates. A total of 9649 protein groups were quantified with an average of 8380 ± 112 and 9022 ± 39 proteins for the MC38 implanted CRC and control mice, respectively (supplemental Table S5). Quantitative proteomic comparison was performed by normalization to correct the batch effect of each sample (supplemental Fig. S8) followed by two-sided Welch’s *t* test (*p*-value < 0.05), yielding 2741 DEPs across the MLNs of the MC38 implanted and control mice (supplemental Table S6). GO category analysis revealed that 348 DEPs are linked to immune functions. The unsupervised cluster heatmap shows the distinct profiles of these immunological DEPs between the MLNs from MC38 implanted and control mice (Fig. 6*A*). The volcano plot reveals the statistically significant immunological DEPs (Fig. 6*B*). For example, TCF7 protein, a transcriptional activator that promotes Th2 cell differentiation (48), shows significant downregulation in MLNs from CRC mice, suggesting an antitumorigenic immune response. In addition, we also identified an upregulated expression of apolipoprotein B mRNA-editing enzyme 3 (APOBEC3) which has been reported to be involved in innate and adaptive immunity (49, 50), and its induced mutagenesis is closely correlated with the response to immunotherapy (20), including CRC (51).

Pathway analysis of the upregulated DEPs against the KEGG immune pathway database using STRING (v12.0) enriched proteins involved in the hematopoietic cell lineage, intestinal immune network for immunoglobulin A production, NOD-like receptor signaling, and complement and coagulation cascades (supplemental Fig. S9). The comparison shows that more immunological DEPs are downregulated and enriched in adaptive immune signaling pathways, such as C-type lectin receptor signaling pathway, chemokine signaling pathway, and toll-like receptor signaling pathway (Fig. 6*C*). In addition, T cell-related pathways, including T cell receptor signaling, Th1 and Th2 cell differentiation, and Th17 cell differentiation, are also among the most significantly downregulated pathways. Notably, further validation by FACS analysis reveals that CD3E, the lineage marker for T cells, showed consistent downregulated expression in the MLNs of MC38 CRC model compared to the downregulated expression by proteomics profiling (Fig. 6, *D* and *E*), suggesting a decrease of the T cell population. Our results reveal an immunosuppressive status of MLNs from MC38 cells inoculated CRC mice, consistent with the reduced percentage of tumor-infiltrating immune cell populations measured by flow cytometry. Lastly, key pathways for other immune cells, including B cell receptor signaling for B cells, Fc gamma receptor-mediated phagocytosis for MPs, and NK cell mediated cytotoxicity for NK cells, were also enriched in the downregulated pathways. With the capability of our libDIA approach to illuminate the immune landscape of the tissue microenvironment, these results may imply a decrease of the T cell populations and adaptive immune cell signaling pathways within the MLNs of MC38 cell recipients.

## Discussion

MS-based proteomic analysis offers a systematic view of the molecular features of the immune microenvironment and may provide new opportunities to identify putative immunomodulatory interventions for tumor immunotherapy (24). However, deep profiling of TME of the biopsy specimens is often limited by limited tissue amount and the highly dynamic and complex nature of heterogeneity. Aiming at deep and accurate immunoproteomics profiling, we generated six immune cell subtype-specific spectral libraries comprising 7815 distinct proteins with ∼50,000 sorted cells from murine MLNs. It is particularly noteworthy given that the libraries were constructed using low cell numbers comparable to microscale samples, *i.e.* μg of tissue, to achieve optimal sensitivity and maximize identification coverage for microscale samples (35). Characterization of the low immune cell populations often adapted the strategy of cell sorting from large sample amounts to obtain a sufficient number of immune cells. In our study, for example, tumors from the CRC model have small percentages of immune cells, ranging from 0.25% to 3% (supplemental Fig. S6*B*). In contrast to such conventional strategy, we adapted a single-shot libDIA workflow for high performance tissue profiling without cell sorting and peptide fractionation. We demonstrated that such immune cell-enriched libraries significantly improved sensitivity and coverage of the tissue immune landscape, enabling the identification of many important immune cell-activating signaling proteins at low abundance, such as BLNK, ZAP70, as well as Food and Drug Administration-approved druggable targets, PI3K and AKT, through efficient single-shot DIA-MS. While some of these low-abundance markers, such as PI3K and AKT are commonly detected in conventional proteomic datasets, they cannot be reliably detected in very small sample inputs, such as microgram samples derived from clinical tissue biopsies where sample amounts are typically limited. Therefore, the exclusive identification of these low-abundance markers by our method can be attributed to the high peptide spectral similarity between the microscale sample size (1 μg of tumor tissue) and the immune cell proteome constructed with microscale immune cells. To the best of our knowledge, this is the first report using as low as 1 μg tissue protein as starting material for microscale tissue proteomics to quantify >7000 proteins, which overcomes the constraints of limited sample size and illuminates the tissue immune microenvironment. We believe that the established spectral library, made publicly accessible, can be adapted by other research groups to enhance immune proteomics profiling performance using animal models. In the near future, our goal is to implement the microscale sample processing protocol from mice to humans to generate human immune cell mass spectral libraries that may facilitate deep tumor profiling from patients using the DIA approach. By the immune cell-specific differential expression, a computation algorithm can be designed to explore the heterologous immune cell activation status.

The spectral libraries established in this study include six primary immune cell subtypes, CD8^+^ T and CD4^+^ T lymphocytes, B lymphocytes, NK cells, DCs, and MPs. The size of the library varied in terms of the protein/peptide numbers, likely due to the varying cell sizes across different cell types, as well as variations in sample preparation efficiency. By comparison with several publicly accessible databases and literatures, we show that the immune cell-enriched library has high coverage of immune proteome and cell subtype-specific proteins, such as 971 proteins associated with the immune system annotated by the GO database, and substantial overlap in the key immune cell pathways in the KEGG database. The specificity and fidelity of our immune cell subtype datasets were also confirmed through comparisons with the validated transcriptomic and proteomic immune cell datasets in literature. Immune cell subtype datasets showed high agreement with the human genome-wide RNA expression profiles of single-cell immune cell types from the HPA (30, 31), despite the expected differences between mouse and human species. At the proteomics level, an average of 80% of each immune cell subtype-specific library overlapped with the homologs of the corresponding human cell subtype proteome from the ImmProt database (22). Collectively, the high consistency between our dataset and previously reported human transcriptomes and proteomes indicated the specificity of our immune cell subtype-specific libraries. In addition, canonical markers of each cell subtype were found aligned with the known expression patterns. On top of that, correlation analysis of the subtype proteome supported the cell lineage in immunology, demonstrating that our immune cell subtype proteomic data recapitulated known aspects of the immune system. These data further suggested that the immune cell-enriched library may serve as a valuable resource to enable deep proteomics analysis for the immune landscape in microscale tissue samples. Our method also provides the flexibility to use a specific cell subtype library or a combination of multiple libraries.

TILs correlate with the number and size of surrounding LNs in patients with CRC and reflect the quality of the antitumor immune responses (52, 53, 54). In this study, we use libraries with multiple immune cell subtypes to improve the coverage of immuno-proteins with low abundance in MC38 cell-inoculated colon tissues. Our identification of immune cell activation proteins, such as ZAP70, LCK, and BLNK indicates an extensive immune cell response in the TME of CRC mice under the condition that the percentage of CD45^+^ cells is less than 3%, representing a characteristic of "cold" tumors (46). The increased coverage of BTK, BLNK, and BANK1 effectively contributes to the monitoring of B cell activation, differentiation, and survival (55, 56). Identification of ZAP70, Src family kinases LCK, and proto-oncogene tyrosine-protein kinase (FYN) is also critical for detecting the activation and development of CD4^+^ and CD8^+^ T cells in the TME of the CRC model (57, 58). Since cold tumors have a significant negative impact on clinical efficacy in cancer patients (59, 60, 61), effective differentiation between “hot” and “cold” tumors is critical for predicting the efficacy of immunotherapy. Our study introduces a novel, high-resolution method for analyzing the tumor immune microenvironment that overcomes the limited resolution of immune cell activity at low infiltration abundance, and allows us to identify the trend of antitumor immunity, which could improve the effectiveness of personalized immunotherapy.

In addition to TILs, sentinel LNs support systemic antitumor immune surveillance and promote the efficacy of immune checkpoint therapy. For example, lymphatic transport facilitates antigen presentation to CD8^+^ T lymphocytes in sentinel LN, and priming tumor-associated immunity (62, 63, 64). Meanwhile, sentinel LNs, including MLNs, are the most common clinically relevant metastatic foci of CRC (65). The immune cell-enriched libDIA showed different expression profiles of immune markers in the MLNs of mice with CRC tumors compared to control samples, including upregulation of the intestinal immune network for immunoglobulin A production, NOD-like receptor signaling, and innate immune signaling pathways. In contrast, signaling pathways correlated with adaptive immunity were significantly downregulated, such as T cell receptor signaling, C-type lectin receptor signaling, Fc gamma receptor-mediated phagocytosis, and T cell differentiation pathways, including Th1, Th2, and Th17 cell differentiation signaling. Supported by the validation of the reduction of the T cell frequency in the MLNs of CRC mice, our method demonstrates an immunosuppressive response in the early stage of tumor development. Furthermore, our data show that innate immunity is upregulated. Since innate immunity is essential for the onset and maintenance of adaptive immunity, and orchestrates tumor progression (66), we expect to further use the MLN samples from CRC mice with different metastatic stages to illustrate the scenario of antitumor immunosurveillance in CRC mice from early to advanced stage.

In summary, we report an immune cell-enriched libDIA method that capitalizes on providing deep immune-proteomic analysis with high sensitivity for microscale tissues. Label-free quantitative proteome profiling of the immune microenvironment can be achieved by integrating the immune cell subtype-specific libraries with a single-shot libDIA approach. With the demonstration on microscale (1 μg) of tissue, our method can be easily adapted for deep sensitivity and coverage of immune landscape profiling. The application to MLNs from MC38 recipients showcased its capability for in-depth profiling of immune heterogeneity and discovering low-abundance immune markers, thereby enhancing our understanding of the complexity of the tumor immune microenvironment. This approach together with the established spectral libraries serves as a generic platform for future investigations into the intricate dynamics of immune cell subtypes in various physiologic and pathologic scenarios.

## Data Availability

The mass spectrometry raw files, reference spectral libraries, library protein lists, search results, and protein identification lists in this study are available *via* ProteomeXchange with the identifier PXD048670 and jPOST with the identifier JPST002410 (67).

## Supplemental data

This article contains supplemental data.

## Conflict of interest

The authors declare no competing interests.
