# Supplementary material for: Enhanced Proteomic Coverage in Tissue Microenvironment by Immune Cell Subtype Library-Assisted DIA-MS
Source: Mol Cell Proteomics. 2024 May 27;23(7):100792. doi: 10.1016/j.mcpro.2024.100792 (PMC11260568; doi:10.1016/j.mcpro.2024.100792)
Supplement: supplemental Figures S1–S9 [file mmc5.pptx]

## Slide 1
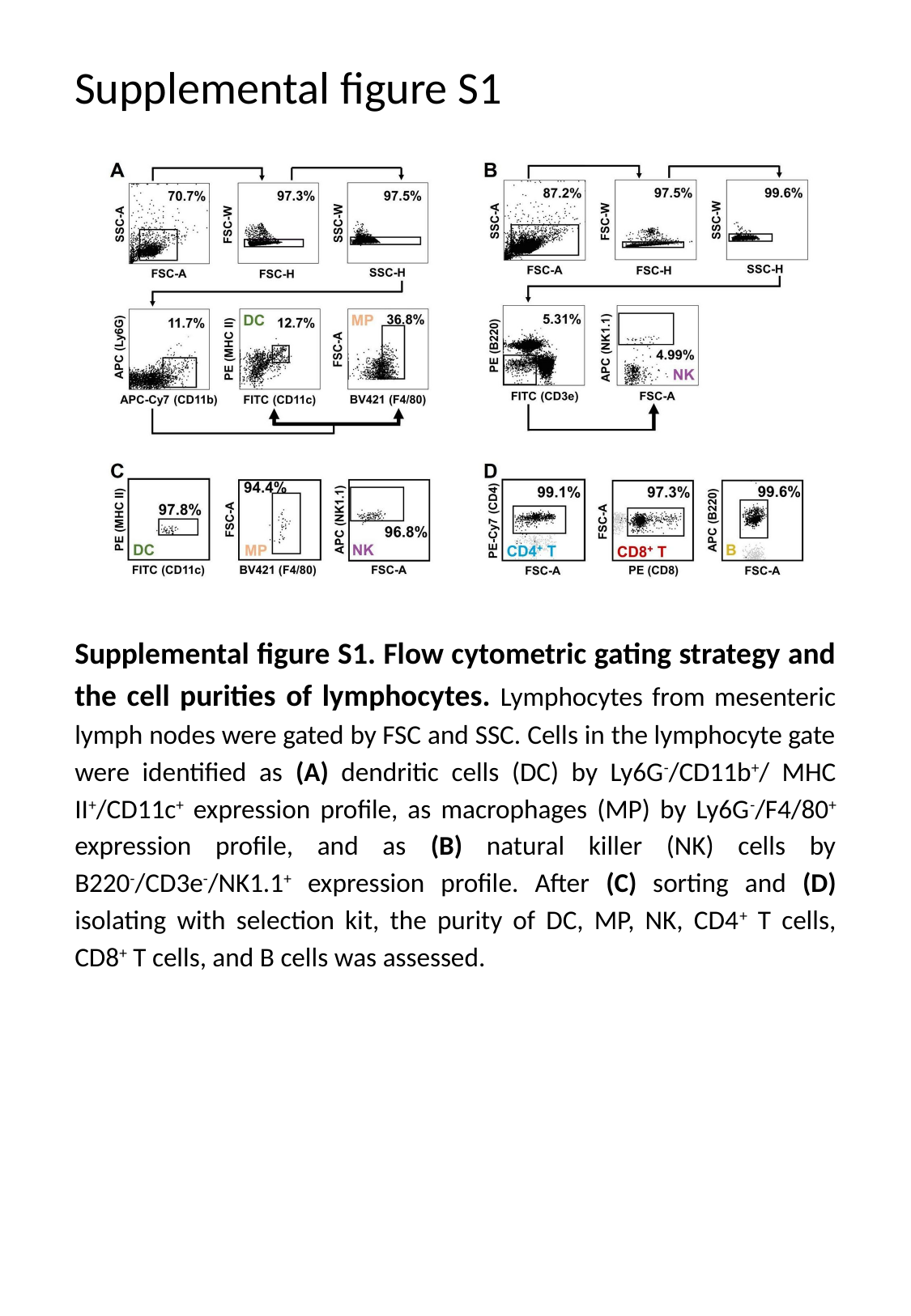

# Supplemental figure S1
Supplemental figure S1. Flow cytometric gating strategy and the cell purities of lymphocytes. Lymphocytes from mesenteric lymph nodes were gated by FSC and SSC. Cells in the lymphocyte gate were identified as (A) dendritic cells (DC) by Ly6G-/CD11b+/ MHC II+/CD11c+ expression profile, as macrophages (MP) by Ly6G-/F4/80+ expression profile, and as (B) natural killer (NK) cells by B220-/CD3e-/NK1.1+ expression profile. After (C) sorting and (D) isolating with selection kit, the purity of DC, MP, NK, CD4+ T cells, CD8+ T cells, and B cells was assessed.

## Slide 2
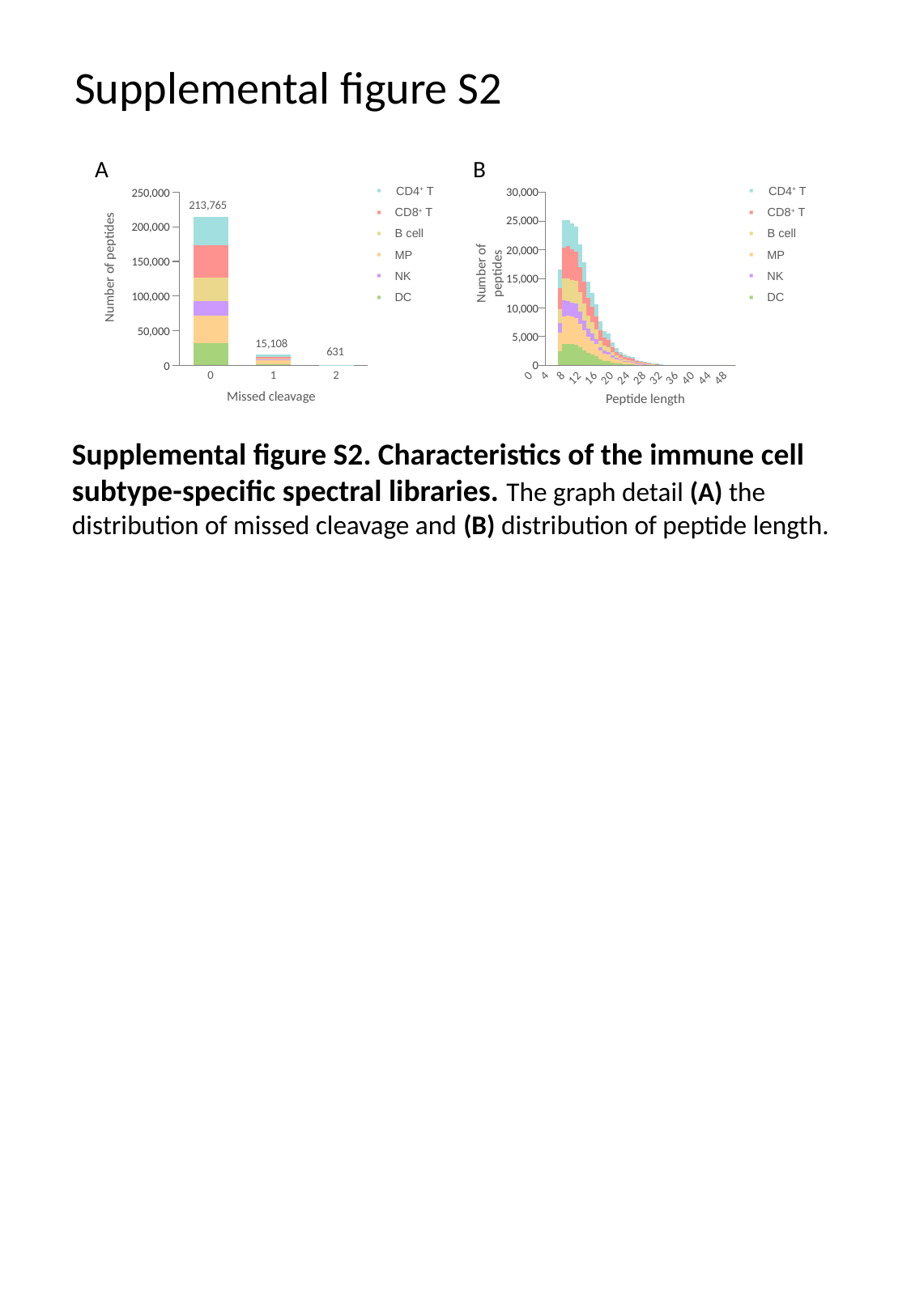

# Supplemental figure S2
A
B
### Chart
| Category | DC | MP | NK | B | CD8 | CD4 |
|---|---|---|---|---|---|---|
| 0 | 31653.0 | 39692.0 | 20905.0 | 33811.0 | 47741.0 | 39963.0 |
| 1 | 1658.0 | 5370.0 | 986.0 | 1470.0 | 2418.0 | 3206.0 |
| 2 | 53.0 | 336.0 | 33.0 | 31.0 | 36.0 | 142.0 | 213,765
Number of peptides
 15,108
 631
Missed cleavage
### Chart
| Category | DC | MP | NK | B | CD8 | CD4 |
|---|---|---|---|---|---|---|
| 0 | None | None | None | None | None | None |
| 1 | None | None | None | None | None | None |
| 2 | None | None | None | None | None | None |
| 3 | None | None | None | None | None | None |
| 4 | None | None | None | None | None | None |
| 5 | None | None | None | None | None | None |
| 6 | None | None | None | None | None | None |
| 7 | 2445.0 | 3213.0 | 1647.0 | 2428.0 | 3628.0 | 3233.0 |
| 8 | 3762.0 | 4742.0 | 2783.0 | 3849.0 | 5317.0 | 4762.0 |
| 9 | 3755.0 | 4859.0 | 2530.0 | 3890.0 | 5609.0 | 4518.0 |
| 10 | 3734.0 | 4709.0 | 2417.0 | 3929.0 | 5336.0 | 4424.0 |
| 11 | 3610.0 | 4599.0 | 2475.0 | 3925.0 | 5089.0 | 4369.0 |
| 12 | 3110.0 | 4074.0 | 2089.0 | 3355.0 | 4438.0 | 3922.0 |
| 13 | 2601.0 | 3484.0 | 1749.0 | 2876.0 | 3815.0 | 3380.0 |
| 14 | 2144.0 | 2840.0 | 1347.0 | 2281.0 | 3152.0 | 2723.0 |
| 15 | 1824.0 | 2460.0 | 1189.0 | 1966.0 | 2747.0 | 2325.0 |
| 16 | 1537.0 | 2125.0 | 901.0 | 1627.0 | 2333.0 | 2032.0 |
| 17 | 1044.0 | 1543.0 | 606.0 | 1144.0 | 1763.0 | 1483.0 |
| 18 | 808.0 | 1267.0 | 461.0 | 863.0 | 1377.0 | 1132.0 |
| 19 | 746.0 | 1136.0 | 440.0 | 789.0 | 1279.0 | 1086.0 |
| 20 | 491.0 | 899.0 | 304.0 | 564.0 | 927.0 | 811.0 |
| 21 | 378.0 | 688.0 | 205.0 | 424.0 | 666.0 | 657.0 |
| 22 | 316.0 | 552.0 | 168.0 | 304.0 | 512.0 | 514.0 |
| 23 | 253.0 | 440.0 | 143.0 | 261.0 | 420.0 | 404.0 |
| 24 | 198.0 | 385.0 | 124.0 | 208.0 | 380.0 | 371.0 |
| 25 | 170.0 | 338.0 | 103.0 | 197.0 | 312.0 | 301.0 |
| 26 | 94.0 | 223.0 | 68.0 | 112.0 | 235.0 | 196.0 |
| 27 | 84.0 | 217.0 | 48.0 | 97.0 | 195.0 | 160.0 |
| 28 | 69.0 | 144.0 | 37.0 | 58.0 | 153.0 | 128.0 |
| 29 | 50.0 | 112.0 | 20.0 | 48.0 | 129.0 | 93.0 |
| 30 | 48.0 | 89.0 | 26.0 | 47.0 | 118.0 | 82.0 |
| 31 | 32.0 | 76.0 | 11.0 | 27.0 | 89.0 | 65.0 |
| 32 | 13.0 | 39.0 | 9.0 | 11.0 | 59.0 | 31.0 |
| 33 | 9.0 | 23.0 | 6.0 | 10.0 | 22.0 | 14.0 |
| 34 | 12.0 | 34.0 | 6.0 | 9.0 | 26.0 | 27.0 |
| 35 | 2.0 | 12.0 | 1.0 | 2.0 | 10.0 | 16.0 |
| 36 | 10.0 | 14.0 | 2.0 | 3.0 | 17.0 | 13.0 |
| 37 | 3.0 | 12.0 | 2.0 | 2.0 | 6.0 | 8.0 |
| 38 | 3.0 | 10.0 | None | 1.0 | 8.0 | 7.0 |
| 39 | 1.0 | 10.0 | 1.0 | None | 7.0 | 5.0 |
| 40 | 4.0 | 8.0 | 3.0 | 2.0 | 7.0 | 7.0 |
| 41 | 1.0 | 9.0 | 1.0 | 1.0 | 1.0 | 4.0 |
| 42 | None | 4.0 | None | 1.0 | 4.0 | 2.0 |
| 43 | 1.0 | 3.0 | 1.0 | 1.0 | 5.0 | 2.0 |
| 44 | None | 1.0 | None | None | 1.0 | None |
| 45 | 1.0 | 1.0 | 1.0 | None | None | 1.0 |
| 46 | None | 1.0 | None | None | 2.0 | 1.0 |
| 47 | None | 1.0 | None | None | None | 1.0 |
| 48 | 1.0 | None | None | None | 1.0 | 1.0 |
| 49 | None | 1.0 | None | None | None | None |
| 50 | None | 1.0 | None | None | None | None |Number of peptides
Peptide length
CD4+ T
■
CD8+ T
■
B cell
■
MP
■
NK
■
DC
■
CD4+ T
■
CD8+ T
■
B cell
■
MP
■
NK
■
DC
■
30,000
25,000
20,000
15,000
10,000
5,000
0
250,000
200,000
150,000
100,000
50,000
0
Supplemental figure S2. Characteristics of the immune cell subtype-specific spectral libraries. The graph detail (A) the distribution of missed cleavage and (B) distribution of peptide length.

## Slide 3
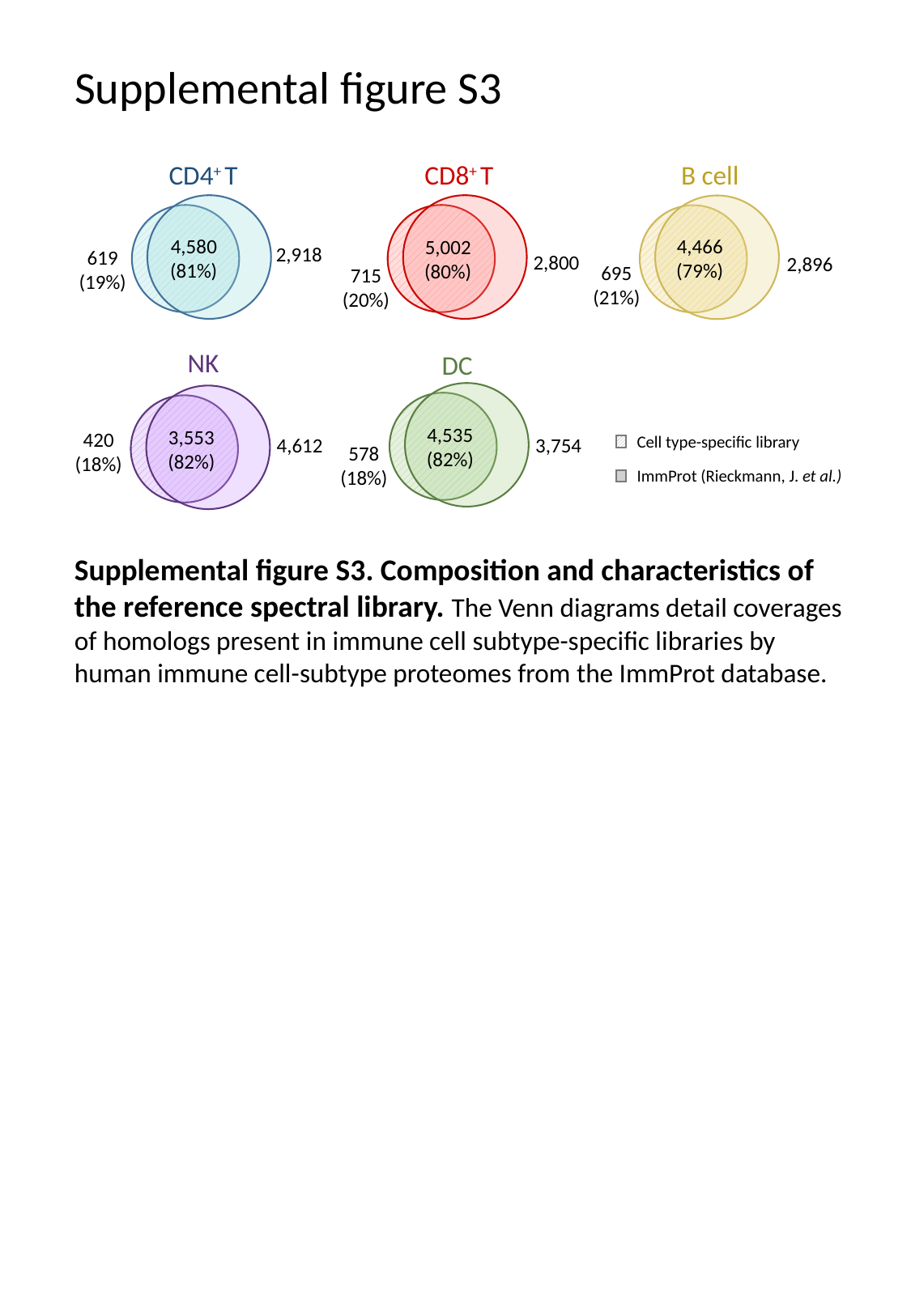

# Supplemental figure S3
B cell
4,466
(79%)
2,896
695
(21%)
CD4+ T
4,580
(81%)
2,918
619
(19%)
CD8+ T
5,002
(80%)
2,800
715
(20%)
NK
3,553
(82%)
420
(18%)
4,612
DC
4,535
(82%)
3,754
578
(18%)
Cell type-specific library
ImmProt (Rieckmann, J. et al.)
Supplemental figure S3. Composition and characteristics of the reference spectral library. The Venn diagrams detail coverages of homologs present in immune cell subtype-specific libraries by human immune cell-subtype proteomes from the ImmProt database.

## Slide 4
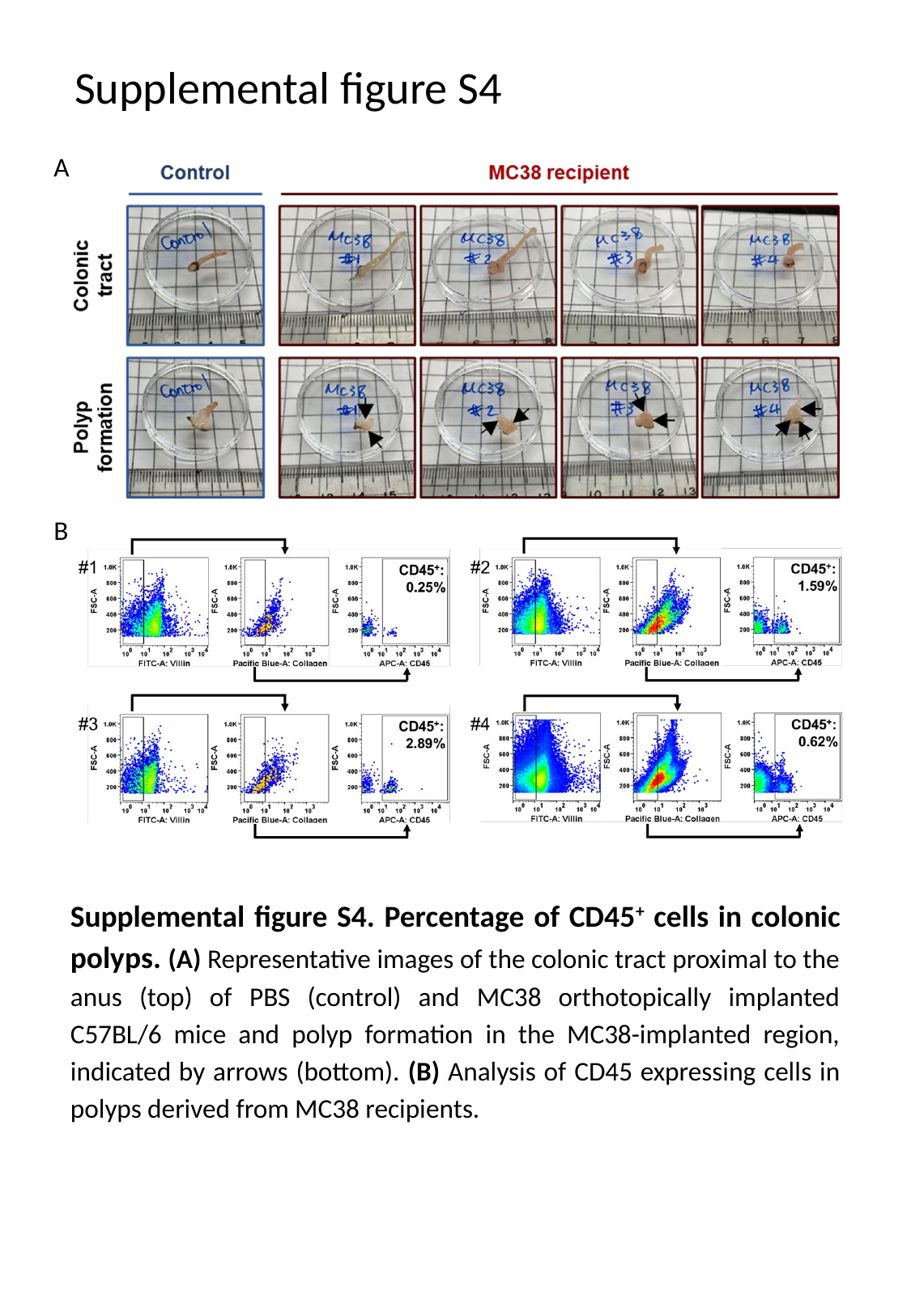

# Supplemental figure S4
A
B
Supplemental figure S4. Percentage of CD45+ cells in colonic polyps. (A) Representative images of the colonic tract proximal to the anus (top) of PBS (control) and MC38 orthotopically implanted C57BL/6 mice and polyp formation in the MC38-implanted region, indicated by arrows (bottom). (B) Analysis of CD45 expressing cells in polyps derived from MC38 recipients.

## Slide 5
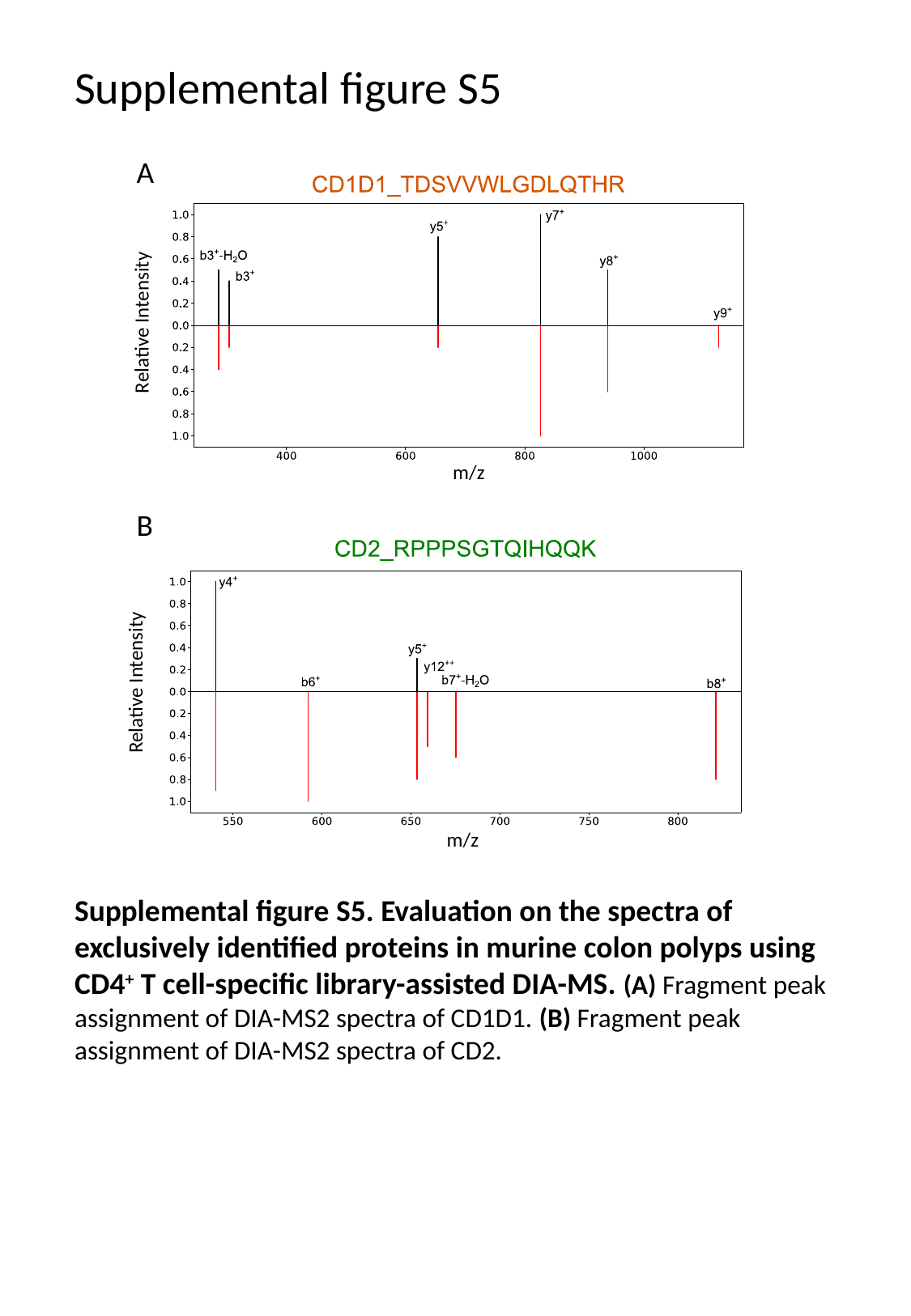

# Supplemental figure S5
A
Relative Intensity
m/z
B
Relative Intensity
m/z
Supplemental figure S5. Evaluation on the spectra of exclusively identified proteins in murine colon polyps using CD4+ T cell-specific library-assisted DIA-MS. (A) Fragment peak assignment of DIA-MS2 spectra of CD1D1. (B) Fragment peak assignment of DIA-MS2 spectra of CD2.

## Slide 6
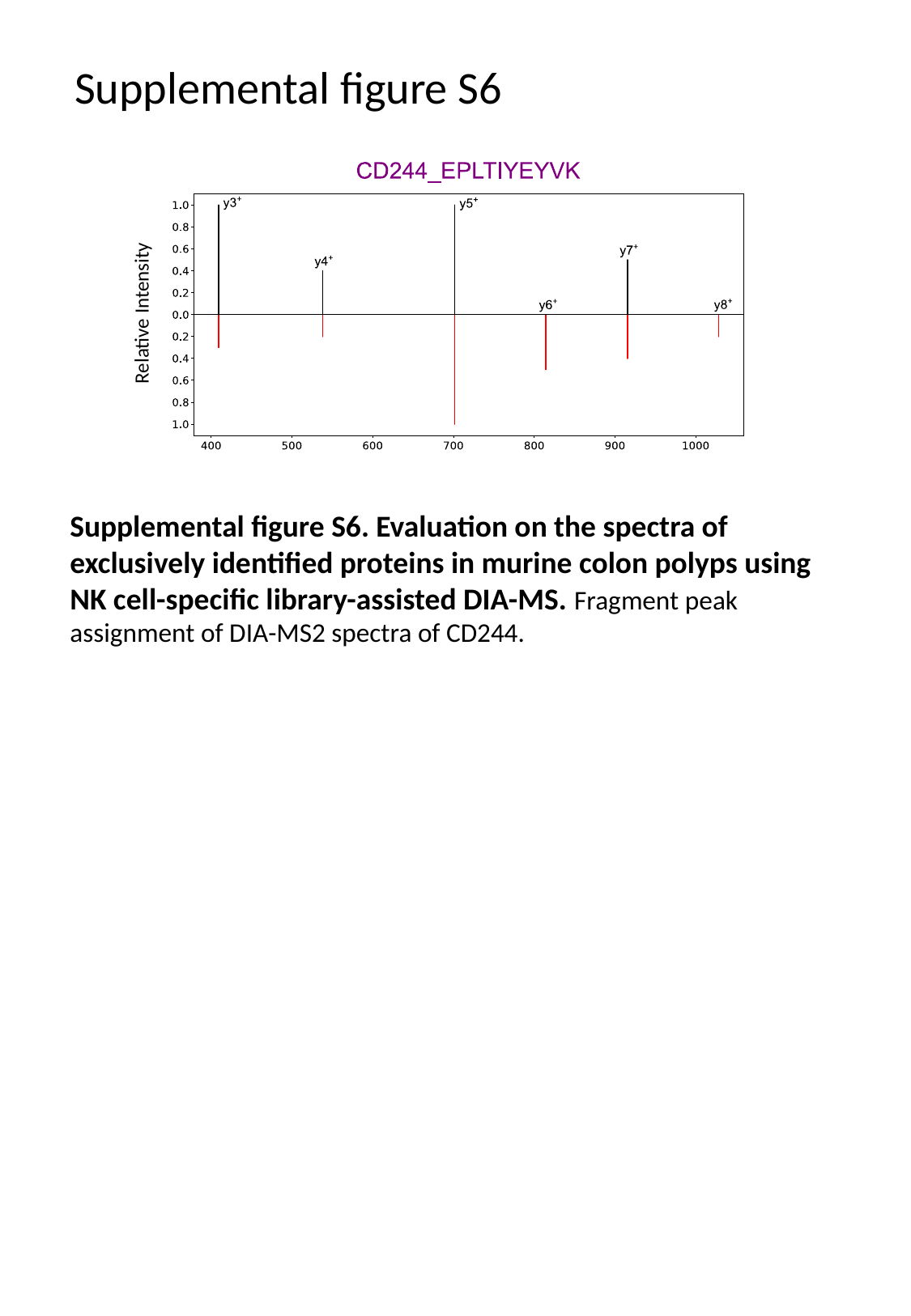

Supplemental figure S6
Relative Intensity
Supplemental figure S6. Evaluation on the spectra of exclusively identified proteins in murine colon polyps using NK cell-specific library-assisted DIA-MS. Fragment peak assignment of DIA-MS2 spectra of CD244.

## Slide 7
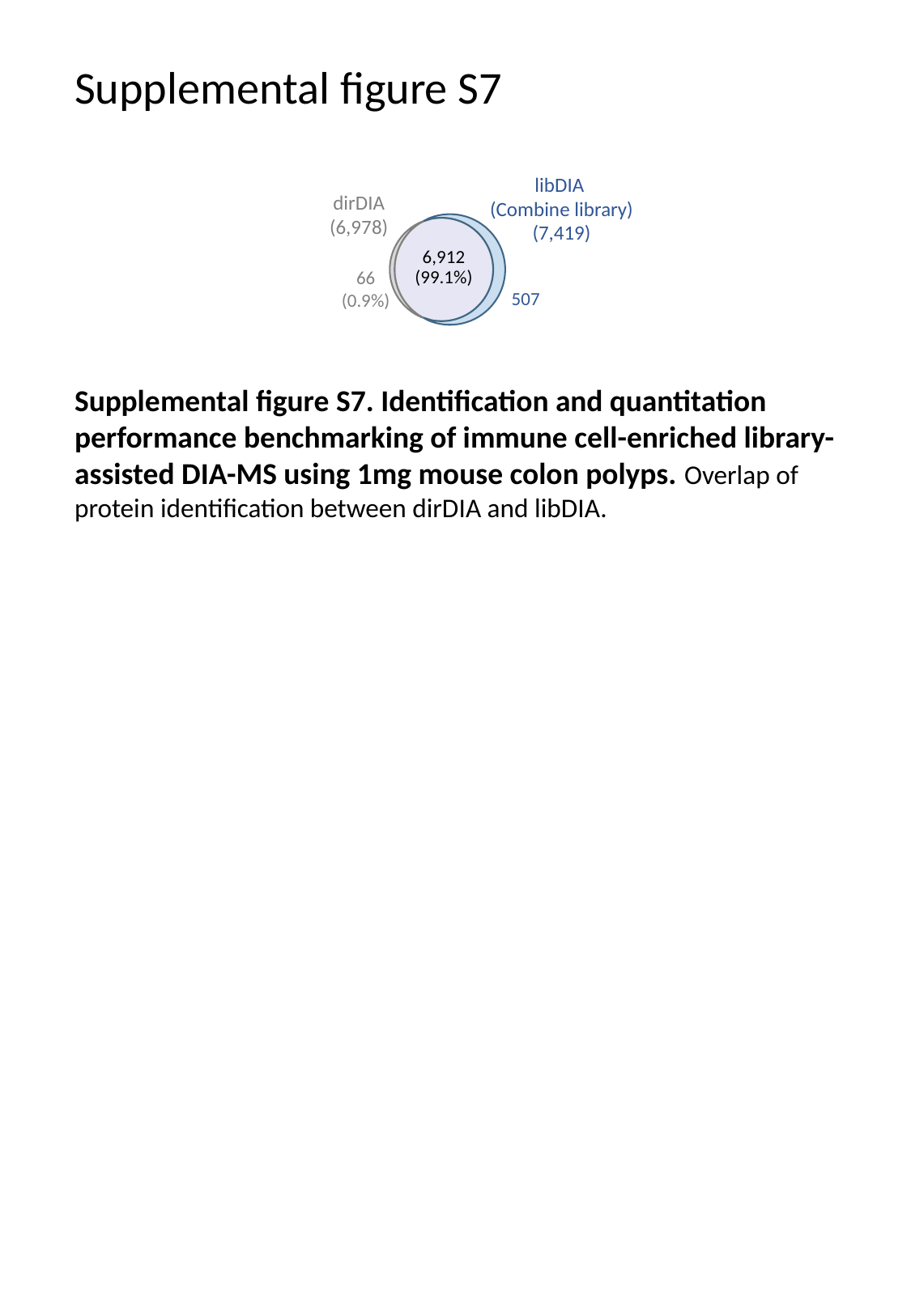

Supplemental figure S7
libDIA
(Combine library)
(7,419)
dirDIA
(6,978)
6,912
(99.1%)
66
(0.9%)
507
Supplemental figure S7. Identification and quantitation performance benchmarking of immune cell-enriched library-assisted DIA-MS using 1mg mouse colon polyps. Overlap of protein identification between dirDIA and libDIA.

## Slide 8
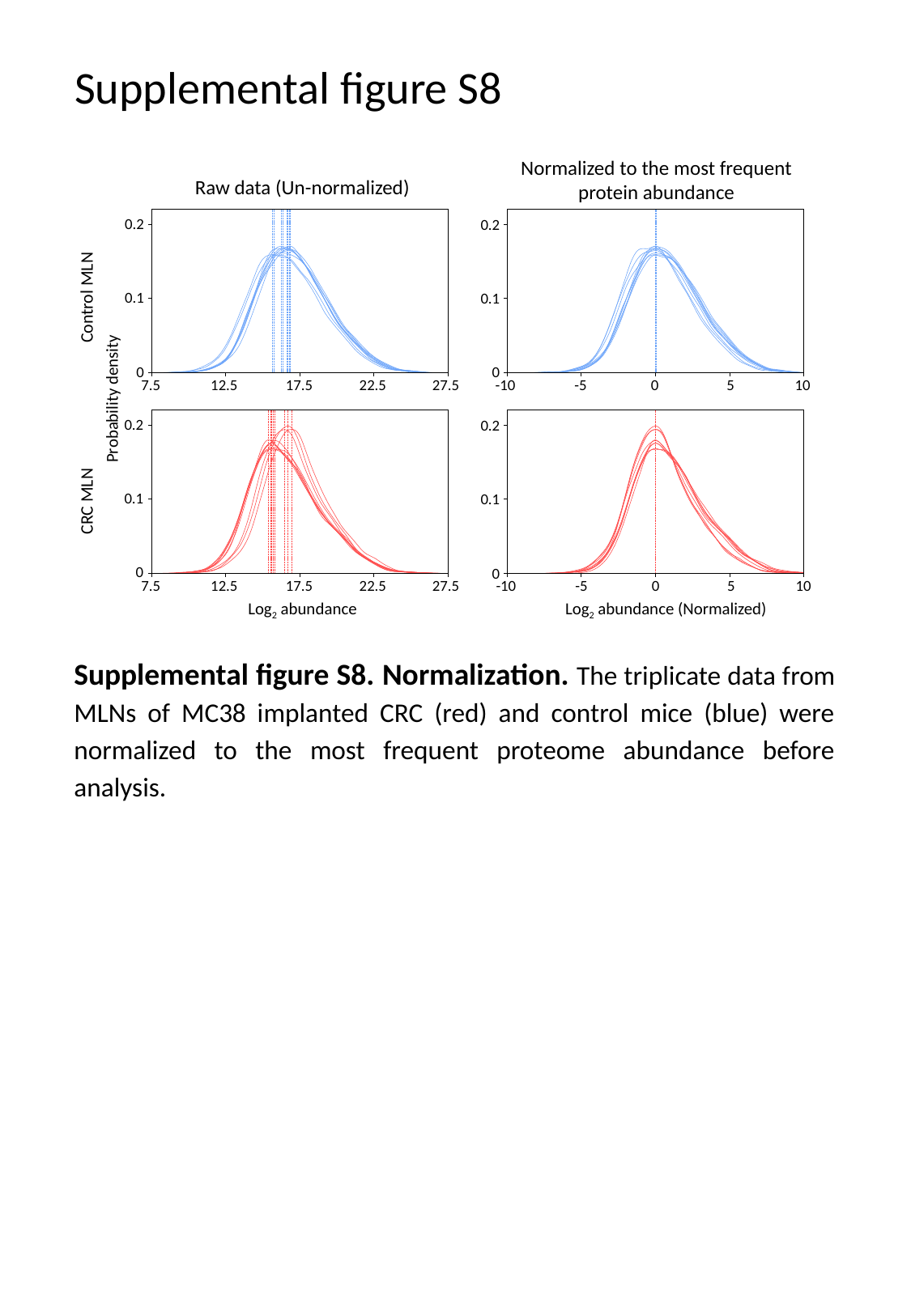

Supplemental figure S8
Normalized to the most frequent protein abundance
Raw data (Un-normalized)
0.2
0.1
0
0.2
0.1
0
Control MLN
7.5
12.5
17.5
22.5
27.5
-10
-5
0
5
10
Probability density
0.2
0.1
0
0.2
0.1
0
CRC MLN
7.5
12.5
17.5
22.5
27.5
-10
-5
0
5
10
Log2 abundance
Log2 abundance (Normalized)
Supplemental figure S8. Normalization. The triplicate data from MLNs of MC38 implanted CRC (red) and control mice (blue) were normalized to the most frequent proteome abundance before analysis.

## Slide 9
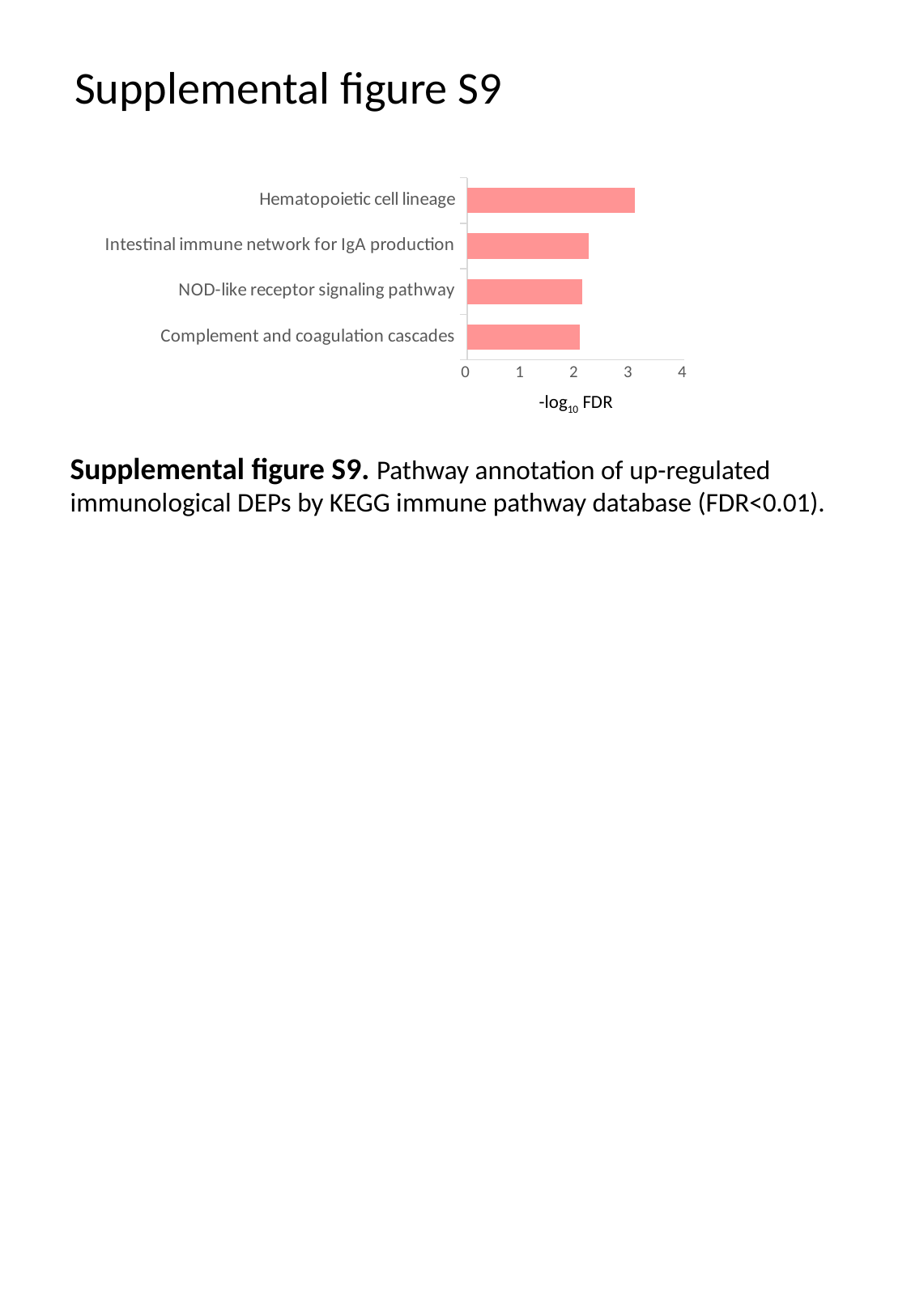

Supplemental figure S9
### Chart
| Category | |
|---|---|
| Complement and coagulation cascades | 2.0757207139381184 |
| NOD-like receptor signaling pathway | 2.1191864077192086 |
| Intestinal immune network for IgA production | 2.2441251443275085 |
| Hematopoietic cell lineage | 3.096910013008056 |-log10 FDR
Supplemental figure S9. Pathway annotation of up-regulated immunological DEPs by KEGG immune pathway database (FDR<0.01).
